# Inhibition of MCL-1 to eliminate senescent cells and mitigate renal fibrosis in aristolochic acid nephropathy

**DOI:** 10.1038/s41419-025-08268-7

**Published:** 2025-11-26

**Authors:** Peng Gao, Schrodinger Cenatus, Nathalie Henley, Vincent Pichette, Frédérick A. Mallette, Jonatan Barrera-Chimal, Casimiro Gerarduzzi

**Affiliations:** 1 Department of Pharmacology and Physiology, Faculty of Medicine, University of Montreal, Montreal, QC Canada; 2 Maisonneuve-Rosemont Hospital Research Center, Center affiliated with the University of Montreal, Montreal, QC Canada; 3 Department of Biochemistry and Molecular Medicine, Faculty of Medicine, University of Montreal, Montreal, QC Canada; 4 Department of Medicine, Faculty of Medicine, University of Montreal, Montreal, QC Canada; 5 Division of Nephrology, Maisonneuve-Rosemont Hospital, Montreal, QC Canada

**Keywords:** Chronic kidney disease, Chronic inflammation

## Abstract

The role of tubular epithelial cells (TEC) senescence in the progression from acute kidney injury (AKI) to chronic kidney disease (CKD) remains debated due to the complexity of senescent cell populations and their pro-survival mechanisms. To directly assess the contribution of TEC senescence to AKI-to-CKD progression, we employed an aristolochic acid nephropathy (AAN) mouse model. Here, we demonstrated that AAI-induced DNA damage specifically drives TEC senescence during AKI-to-CKD progression. Concomitant with the emergence of senescence, immunofluorescence staining revealed the expression of anti-apoptotic proteins, including BCL-2, BCL-xL, and MCL-1, within KIM1⁺ tubules—a marker of tubular injury. To further characterize these senescent cells, we integrated this model with snRNA-Seq data and identified a distinct population of KIM1^+^ senescent TEC exhibiting resistance to apoptosis through upregulation of pro-survival proteins such as MCL-1, BCL-2, and BCL-xL. To evaluate the therapeutic potential of targeting these pathways, we treated AAN mice with the MCL-1-specific inhibitor UMI-77 and the senolytic ABT-263 (targeting BCL-2/BCL-xL) during both the acute and late phases. Interestingly, only UMI-77 administration during the acute phase effectively reduced tubular senescence and mitigated fibrosis. In contrast, late-phase treatment had only marginal benefits. Notably, ABT-263 failed to eliminate senescent cells and instead exacerbated fibrosis, suggesting that while senescent TEC relies on pro-survival mechanisms to evade apoptosis, their dependency on specific anti-apoptotic proteins varies. Our study provides a high-resolution molecular framework for understanding TEC senescence and identifies MCL-1 inhibition as a precise and effective therapeutic strategy to prevent AKI-to-CKD progression, with early intervention being critical for therapeutic success.

## Introduction

Acute kidney injury (AKI) is defined as a rapid decline in kidney function, often due to ischemia, toxins, or infections [1], while chronic kidney disease (CKD) is marked by the progressive loss of function in the kidneys over an extended period, which ultimately leads to end-stage renal disease (ESRD) if left untreated [2]. Despite the possibility of recovery, unresolved or recurrent AKI episodes can cause chronic inflammation and tissue damage, eventually leading to CKD [3]. A key pathological feature associated with AKI to CKD transition is kidney fibrosis [4], defined by excessive extracellular matrix (ECM) deposition that disrupts the normal architecture and function of the kidney.

Renal tubular epithelial cells (TEC) are the predominant cell type in the kidney and are essential for maintaining renal function [5]. Due to their high metabolic activity, TEC is particularly susceptible to injury from various stressors [5, 6], which often leads to cellular senescence [7]. Senescence is a state of irreversible cell cycle arrest, typically regulated by the p53/p21 and p16/Rb pathways [8], and is triggered by multiple forms of cellular stress, including DNA damage [8]. Senescent cells exhibit distinct biochemical and functional changes [8], such as resistance to apoptosis, increased activity of senescence-associated β-galactosidase (SA-β-gal), and the secretion of a complex mixture of bioactive molecules collectively termed the senescence-associated secretory phenotype (SASP). SASP includes a wide range of proinflammatory cytokines, growth factors, proteases, and ECM regulators [8]. Among the key upstream regulators of SASP is the NF-κB signaling pathway [8]. Another hallmark of senescent cells is their resistance to apoptosis, which is largely due to the upregulation of anti-apoptotic proteins, such as BCL-2 and BCL-xL [9]. This characteristic has opened avenues for the development of pharmacological agents-known as senolytics-that selectively eliminate senescent cells while sparing normal, healthy ones [9]. By targeting these apoptosis-resistant senescent cells, senolytic therapy has shown promise in alleviating renal fibrosis in CKD [10–13]. The therapeutic rationale stems from the observation that, in CKD, the persistent presence of senescent cells contributes to a chronic inflammatory and profibrotic milieu through the secretion of proinflammatory (e.g., CCL2) and profibrotic (e.g., TGF-β1, PAI-1) factors [12]. This environment promotes the activation and transformation of renal fibroblasts into myofibroblasts (FMT), which drives fibrosis through the overproduction of ECM in the renal interstitium [12, 14].

Although the detrimental role of TEC senescence in CKD has been well recognized, its role in AKI remains controversial [12, 15–17]. This is due to variability in how senescence is induced across different animal models, the potential involvement of other renal cell types, and the timing of senescent cells depletion. To address these challenges, we employed aristolochic acid I (AAI), a nephrotoxicant known to cause DNA damage specifically in TEC via selective uptake through organic anion transporters 1, 3 (OAT1, 3) [18, 19]. While AAI-induced DNA damage is well established, whether this insult is sufficient to elicit a senescence response in TEC remains unclear. Thus, a key objective of this study was to determine whether AAI-induced nephropathy (AAN) constitutes a suitable model for studying TEC senescence in the context of kidney injury. Additionally, unlike other models such as ischemia-reperfusion injury (IRI) or unilateral ureteral obstruction (UUO), which can involve multi-compartmental damage [20], the AAN model allows for a more targeted study of TEC senescence. Furthermore, this model effectively simulates the AKI-to-CKD transition driven by acute tubular injury and fibrosis. Using this model, we further evaluated the therapeutic potential of senolytic strategies by assessing their efficacy in eliminating AAI-induced senescent TEC and mitigating renal fibrosis.

## Results

### Selective accumulation of DNA damage in proximal TEC following AAI exposure

**Fig. 1:**
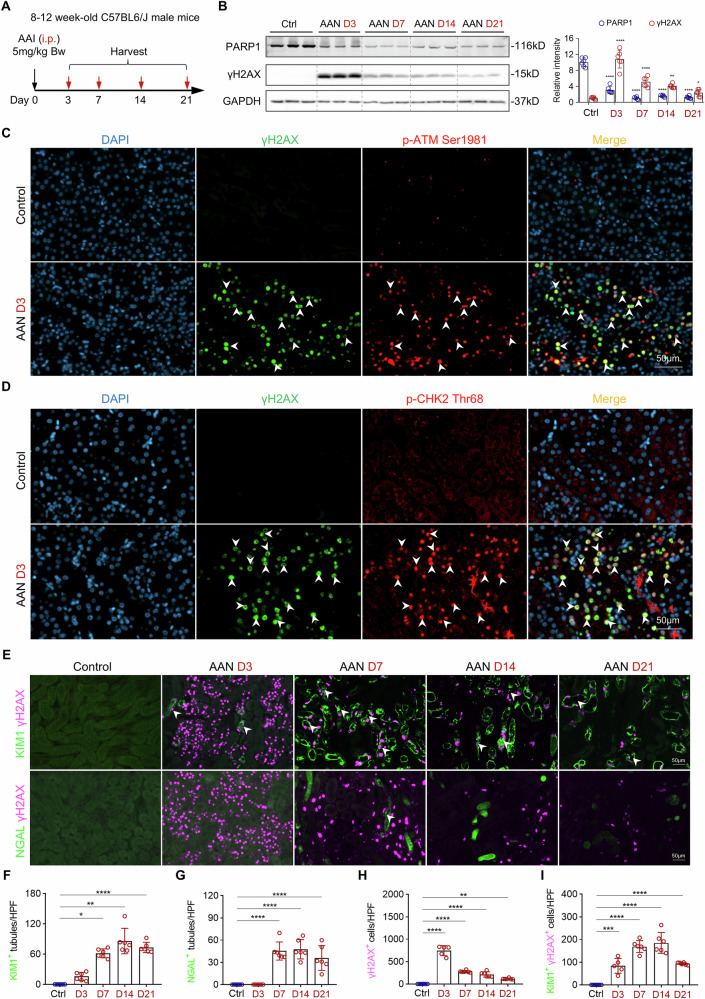
AAI induces tubular epithelial cells injury by direct DNA damage. **A** Experimental design of sample collections from AAN mice. Single dose of 5 mg/kg Bw AAI or Vehicle (DMSO) were injected into male C57BL/6J mice intraperitoneally (i.p.) and sacrificed at D3, D7, D14, and D21. **B** Representative Western blot analysis and quantification of PARP1 and γH2AX of whole kidney lysates. **C** Representative images of γH2AX co-stained with p-ATM Ser 1981 at day 3 after AAI injection. **D** Representative images of γH2AX co-stained with p-CHK2 Thr 68 at day 3 after AAI injection. **E** Representative images of γH2AX co-stained with proximal tubular damage marker (KIM1) and distal nephron damage marker (NGAL) at various time points after AAI injection. **F**–**I** Quantification of KIM1^+^ tubules, NGAL^+^ tubules, γH2AX^+^ cells, and γH2AX^+^ KIM1^+^ tubules per high power field (HPF, x400) in AAN mice. n = 6 for each time point, *p < 0.05, **p < 0.01, ***p < 0.001, ****p < 0.0001.

To induce AAN, male C57BL/6 mice received a single intraperitoneal injection of AAI at a dose of 5 mg/kg Bw, and kidneys were harvested on days 3, 7, 14, and 21 post-injection (Fig. 1A). Once metabolically activated in TEC, AAI forms aristolactam (AL)-DNA adducts that induce DNA damage [21, 22]. In response, cells activate the DNA damage response (DDR), a complex signaling network that coordinates DNA repair and cell-cycle arrest to maintain genomic integrity [23]. However, persistent DDR signaling resulting from irreparable DNA damage can drive cells into senescence [24]. To investigate DDR activation under AAN conditions, we first examined key DNA damage and repair markers. Western blot analysis revealed a time-dependent decrease in poly (ADP-ribose) polymerase 1 (PARP1) (Fig. 1B), a key protein involved in the DNA damage response, particularly in the repair of single-strand breaks (SSBs) as well as apoptosis [25]. This reduction was accompanied by an increase in phosphorylated histone H2AX at serine 139 (γH2AX) levels (Fig. 1B), a well-established marker of DNA double-strand breaks (DSBs) [26], suggesting impaired repair capacity and accumulation of DSBs over time. To further characterize DDR pathway activation, we performed immunofluorescence staining on day 3, when γH2AX expression peaked. Co-staining revealed that phosphorylated ataxia-telangiectasia mutated (ATM) at serine 1981 (p-ATM, Ser 1981) (Fig. 1C) and its downstream effector checkpoint kinase 2 (CHK2) phosphorylated at threonine 68 (p-CHK-2, Thr 68) colocalized with γH2AX foci (Fig. 1D), indicating the activation of the ATM-CHK2 axis in response to DSBs [27]. In parallel, phosphorylation of ataxia telangiectasia and Rad3-related protein (p-ATR) and checkpoint kinase 1 (p-CHK1), typically associated with replication stress and SSBs [27], was also observed at day 3 (Supplementary Fig. 1A, B), albeit with weaker γH2AX colocalization, consistent with their role in sensing SSBs. Next, we assessed the spatial distribution of DNA damage across renal tubule segments. Co-staining of γH2AX with the proximal tubular injury marker kidney injury molecule-1 (KIM1) [28] and the distal tubular injury marker neutrophil gelatinase-associated lipocalin (NGAL) [29] revealed that from day 3 to day 21 post-AAI injection (Fig. 1E), DNA damage was predominantly localized in KIM1^+^ proximal TEC rather than NGAL^+^ distal TEC (Fig. 1F–I). Collectively, these findings indicate that AAI-induced DNA damage is primarily restricted to proximal TEC and leads to sustained activation of DDR pathways, establishing a molecular basis for senescence initiation in this compartment.

### p21 induces senescence in proximal TEC, whereas p16 is expressed in both proximal and distal TEC in AAN

**Fig. 2:**
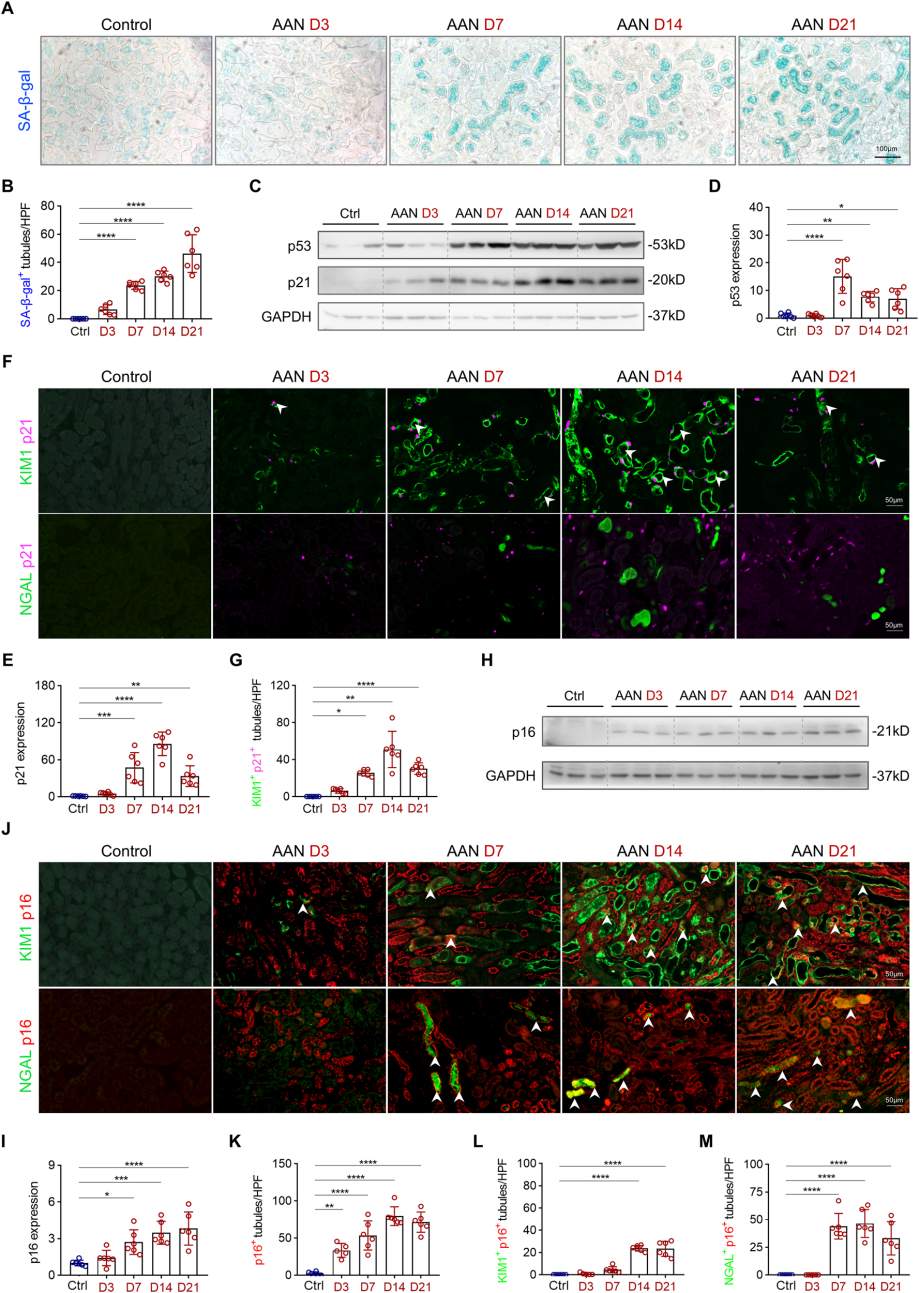
AAN is characterized by senescence induction in tubular epithelial cells. **A**, **B** SA-β-gal staining of kidneys and quantification of SA-β-gal^+^ (blue) tubules per HPF at various time points after AAI injection. **C**–**E** Representative Western blot analysis and quantification of p53 and p21 of whole kidney lysates. **F** Representative images of p21 co-stained with KIM1 and NGAL at various time points after AAI injection. **G** Quantification of p21^+^ KIM1^+^ tubules per HPF in AAN mice. **H**, **I** Representative Western blot analysis and quantification of p16 of whole kidney lysates. **J** Representative images of p16 co-stained with KIM1 and NGAL at various time points after AAI injection. **K**–**M** Quantification of p16^+^ tubules, p16^+^ KIM1^+^ tubules cells, and p16^+^ NGAL^+^ tubules per HPF in AAN mice. n = 6 for each time point, *p < 0.05, **p < 0.01, ***p < 0.001, ****p < 0.0001.

DNA damage is a well-established trigger of cellular senescence [30]. To determine whether AAI-induced DNA damage leads to senescence, we first performed SA-β-gal staining, a well-established marker of senescence [31], on kidney sections from AAI-treated mice (Fig. 2A). A marked increase in staining intensity was observed from day 7 to day 21 post-injection, whereas control kidneys showed minimal staining (Fig. 2B). The DDR involves a cascade of sensors and transducers that activate downstream effectors, such as the p53/p21 axis, leading to cell cycle arrest and the onset of senescence [32]. Western blot analysis revealed early senescence markers p53 and p21 were upregulated as early as day 3 following AAI exposure, peaked around day 14, and remained increased by day 21 (Fig. 2C–E). Notably, immunofluorescence analysis revealed that p21 expression colocalized with the KIM1, but not with the NGAL (Fig. 2F, G), indicating that p21-mediated senescence primarily occurs in injured proximal TEC. In contrast, the expression of p16, a marker of late-stage senescence or maintain senescence [33], became more pronounced at later time points (Fig. 2H, I). Furthermore, p16 staining was observed in both KIM1⁺ and NGAL⁺ TEC (Fig. 2J–M), suggesting a prolonged and spatially extended senescent response during the progression of AKI-to-CKD. Collectively, this temporal and spatial pattern implies that while p21 is primarily involved in initiating cell cycle arrest in response to acute DNA damage, p16 may be induced by persistent DDR signaling and contribute to the long-term maintenance of the senescent phenotype.

### NF-κB activation coincides with SASP upregulation in the kidneys of AAN mice

Given that cellular senescence is not only characterized by stable cell cycle arrest but also by the acquisition of a SASP, which can propagate tissue injury [34], we next investigated whether SASP components are upregulated in the kidneys of AAN mice and examined the underlying regulatory mechanisms. Notably, NF-κB has been identified as a key transcriptional regulator of SASP production [33, 35]. Among its subunits, p50 and p65 play critical roles in orchestrating the complex regulatory network underlying senescence and its associated responses [36, 37]. Western blot analysis revealed a marked increase in p65 protein expression between days 7 and 21 following AAI injection (Supplementary Fig. 2A, B). Immunofluorescence staining further demonstrated that both p50 and phosphorylated p65 (p-p65) colocalized with KIM1 (Supplementary Fig. 2C), suggesting NF-κB activation occurs predominantly in damaged tubules. Moreover, nuclear colocalization of p50 and p-p65 was observed in TEC of AAN mice (Supplementary Fig. 2D), consistent with their transcriptional activity within the nucleus. Concomitantly, the expression of canonical NF-κB-dependent SASP factors-including CXCL1, CCL2, IL-1β, IL-6, PAI-1, and TGF-β1-was upregulated beginning on day 7 and persisted through day 21 (Supplementary Fig. 2E–G). These findings support a central role for the NF-κB signaling pathway in driving SASP gene expression during AAI-induced TEC senescence and highlight its contribution to the sustained proinflammatory and profibrotic milieu during AKI-to-CKD transition.

### AAI-induced senescence is accompanied by upregulation of anti-apoptotic proteins in injured tubules

**Fig. 3:**
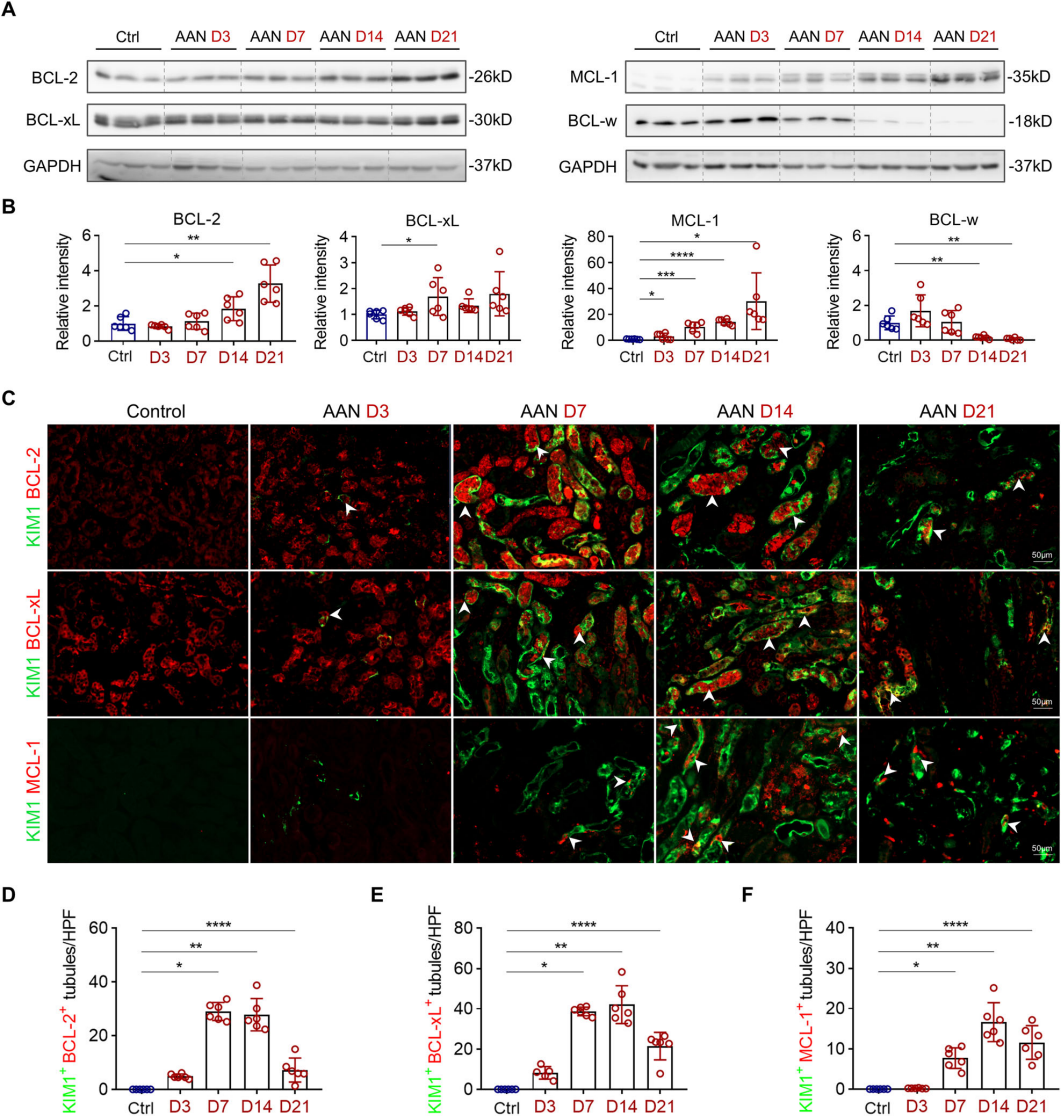
AAI increases the expression of anti-apoptotic proteins in tubular epithelial cells of AAN mice. **A**, **B** Representative Western blot analysis and quantification of anti-apoptotic proteins (BCL-2, BCL-xL, MCL-1, and BCL-w) of whole kidney lysates in the AAN mice. **C** Representative images of KIM1 co-stained with BCL-2, BCL-xL, and MCL-1 at various time points after AAI injection. **D**–**F** Quantification of BCL-2^+^ KIM1^+^ tubules, BCL-xL^+^ KIM1^+^ tubules, and MCL-1^+^ KIM1^+^ tubules per HPF in AAN mice. n = 6 for each time point, *p < 0.05, **p < 0.01, ***p < 0.001, ****p < 0.0001.

Senescent cells remain metabolically active and viable despite having permanently lost their proliferative capacity, largely due to the upregulation of anti-apoptotic proteins from the BCL-2 family [9]. Amongst anti-apoptotic proteins, AAN mice had an increase in renal BCL-2, BCL-xL, and MCL-1 protein expressions over the course of injury, while the opposite occurred for BCL-w (Fig. 3A, B). Furthermore, using KIM1 as a surrogate marker for senescent cells, immunostaining revealed that BCL-2, BCL-xL, and MCL-1 were expressed within KIM1^+^ injured tubules (Fig. 3C–F). Therefore, the tubular expression of various anti-apoptotic BCL-2 family members following AAI injury may be implicated in the survival of senescent TEC.

### AAI-induced tubular injury and interstitial fibrosis mimic the AKI-to-CKD transition

**Fig. 4:**
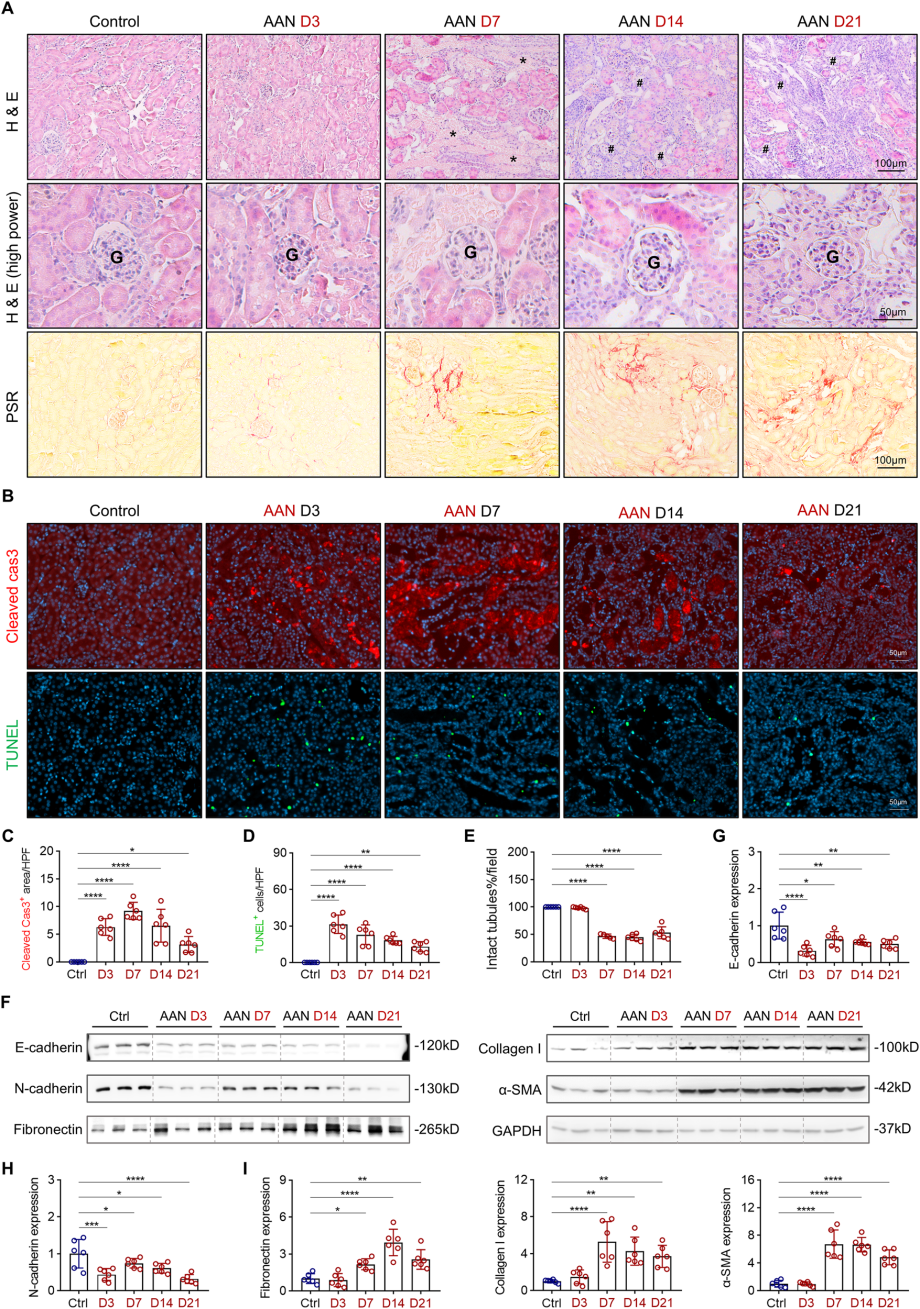
AAI induces acute tubular injury and subsequent tubulointerstitial fibrosis. **A** Representative images of H&E staining (indicating tubular injury) and PSR staining (highlighting collagen I and III deposition) across experimental groups. Asterisks (*) indicate tubular cell death; pound signs (#) indicate atrophic tubules; G denotes glomeruli. **B** Representative images of cleaved caspase-3 and TUNEL staining showing apoptotic cells at different time points following AAI injection. **C** Quantification of cleaved caspase-3 positive cells per HPF. **D** Quantification of TUNEL-positive cells per HPF. **E** Percentage of intact tubules per HPF at various time points after AAI injection. **F** Representative Western blot analysis of tubular epithelial integrity markers (E-cadherin and N-cadherin) and fibrotic markers (fibronectin, collagen I, and α-SMA) in whole kidney lysates. **G**, **H** Quantification of E-cadherin and N-cadherin expression. **I** Quantification of fibronectin, collagen I, and α-SMA expression. n = 6 per time point. Statistical significance: *p < 0.05, **p < 0.01, ***p < 0.001, ***p < 0.0001.

To better characterize the pathological progression of AAI-induced kidney injury, we evaluated the temporal changes in tubular damage and interstitial fibrosis. Compared to control mice, H&E staining revealed an increase in tubular cell death and atrophy over time (Fig. 4A), with no damage observed in the glomeruli (Fig. 4A, middle row). Cleaved caspase-3 immunostaining and TUNEL assays confirmed that apoptosis contributed to tubular cell loss (Fig. 4B–D). As a result, the number of structurally intact tubules decreased significantly over the course of the injury (Fig. 4E). Western blot analysis further validated the loss of tubular integrity, as evidenced by a reduction in E-cadherin and N-cadherin expression beginning as early as day 3 post-injection (Fig. 4F–H). Interstitial fibrosis became apparent from day 7 onward, as shown by PSR staining (Fig. 4A), and was supported by increased expression of fibrotic markers, including fibronectin, collagen I, and α-SMA, as assessed by Western blotting (Fig. 4F, I). These fibrotic markers showed a temporal pattern, rising around day 7, peaking at day 14, and declining slightly by day 21, but still elevated relative to baseline. This sequential pattern of apoptosis and fibrotic remodeling mirrors the dynamic process of the AKI-to-CKD transition, characterized by an initial acute injury phase followed by incomplete resolution and fibrotic remodeling. Notably, the emergence of TEC senescence as early as day 3 (Fig. 2) preceded the onset of fibrosis by day 7, supporting the hypothesis that TEC senescence may drive the development of renal fibrosis.

### AAI induces DNA damage and drives cellular senescence in cultured human tubular epithelial cells

**Fig. 5:**
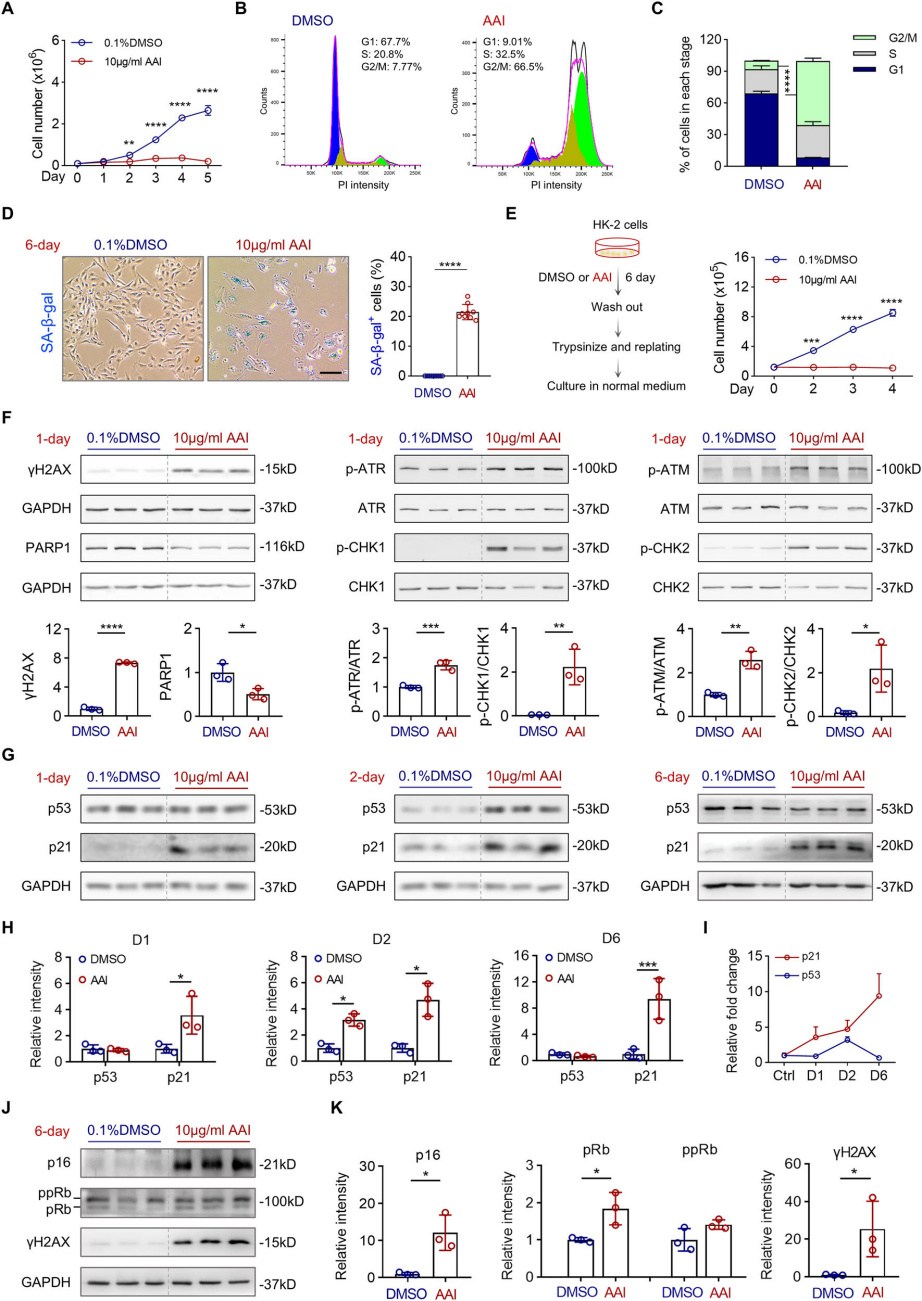
AAI induces DNA damage and senescence in human tubular epithelial cells in vitro. **A** HK-2 cells were treated with 10 μg/mL AAI for 1–5 days, and a trypan blue exclusion assay was used to depict the cellular growth curve. **B** Representative cell cycle analysis by PI staining and flow cytometry in HK-2 after treatment with DMSO or AAI at 10 μg/mL for 6 days. **C** Quantification of the percentage of cells in G2/M, S, and G1 phase. **D** SA-β-gal assay was performed in HK-2 cells following AAI treatment for 6 days, and SA-β-gal^+^ (blue) cells were counted and expressed as percentages of total cell number in randomly selected fields. **E** HK-2 cells were treated with AAI or DMSO for 6 days, harvested by trypsinization, and replated in full growth media without AAI. Cell proliferation was monitored by counting cell numbers on indicated days after re-plating. **F** Western blot was used to analyze the expression of DDR-related proteins (γH2AX, PARP1, p-ATR/ATR, p-CHK1/CHK1, p-ATM/ATM, and p-CHK2/CHK2) and quantification of the above markers. **G**, **H** Western blot was used to analyze the expression of cell cycle regulators (p53 and p21) and quantification of the above markers. **I** Changes of p53 and p21 relative expression over time after AAI treatment. **J**, **K** Western blot analysis was performed to examine cell cycle regulators, including p16, Rb (with the upper band representing the hyperphosphorylated form, ppRb, and the lower band representing the hypophosphorylated form, pRb), and γH2AX, along with quantification of their expression levels. Scale bar: 50μm. n = 3 per condition, *p < 0.05, **p < 0.01, ***p < 0.001, ****p < 0.0001.

Since AAI primarily targets tubular epithelial cells in vivo, we examined its effects in vitro using HK-2 cells, a human proximal tubular epithelial cell line. Treatment with 10 μg/mL AAI led to a marked growth arrest compared to DMSO-treated controls (Fig. 5A). Flow cytometric analysis of DNA content using PI staining revealed a marked accumulation of cells in the G2/M phase (Fig. 5B, C), indicating that AAI induces cell cycle arrest at this checkpoint. SA-β-gal staining was undetectable in DMSO-treated cells but significantly increased in AAI-treated cells over the course of 6 days (Fig. 5D; Supplementary Fig. 3A). Quantitative analysis demonstrated that AAI treatment induced senescence in ~20% of HK-2 cells by day 6. Morphologically, control HK-2 cells maintained a cobblestone appearance characteristic of epithelial monolayers, whereas AAI-treated cells developed enlarged, flattened morphologies with occasional spindle-shaped cells (Supplementary Fig. 3B), consistent with a senescent phenotype. To assess the stability of this growth arrest, AAI-treated cells were trypsinized after 6 days and replated in fresh AAI-free medium. Despite removal of the drug, these cells failed to resume proliferation after an additional 4 days of culture (Fig. 5E), indicating that AAI-induced growth arrest was irreversible once established. We next explored the involvement of the DDR in this senescence induction. 10 μg/mL AAI treatment for just 1 day induced robust γH2AX expression (Fig. 5F). This DNA damage was associated with activation of both the ATR/CHK1 and ATM/CHK2 pathways, as evidenced by increased phosphorylation of these kinases (Fig. 5F). Notably, PARP1 protein levels were markedly reduced following AAI exposure (Fig. 5F), consistent with caspase-mediated degradation, a process often associated with apoptosis. As a key downstream effector of the ATM/CHK2 axis [32], p53 was strongly upregulated after 2 days of AAI treatment, along with its transcriptional target p21 (Fig. 5G, H). Interestingly, while p53 expression declined by day 6, p21 levels remained elevated, suggesting a decoupling of p53-mediated regulation of p21 at later stages of senescence (Fig. 5I). This observation is consistent with reports that p53 downregulation in fully senescent cells may help suppress its pro-apoptotic functions and promote senescent cell survival [38]. By day 6, p16 expression was also significantly increased, along with accumulation of the underphosphorylated form of Rb (pRb) (Fig. 5J, K), both of which are recognized as key regulators of long-term senescence maintenance [33]. Sustained γH2AX expression at this late time point (Fig. 5J, K) further supports the persistence of DNA damage signaling and its contribution to reinforcing the senescent phenotype. Collectively, these findings demonstrate that AAI directly induces cellular senescence in TEC through a DNA damage-mediated mechanism involving both the p53/p21 and p16/Rb pathways.

### AAI-induced senescence is accompanied by epithelial-to-mesenchymal transition (EMT)

EMT is characterized by the loss of epithelial characteristics and the acquisition of mesenchymal features. EMT in injured TEC is recognized as a major contributor to kidney fibrosis [39]. Recent studies have shown that TGF-β1-induced EMT can trigger cell cycle arrest in TEC [40], implicating a possible link between EMT and cellular senescence. Although AAI has previously been reported to induce EMT [18], the interaction between AAI-induced EMT and senescence remains unclear. To explore this relationship, we treated HK-2 cells with AAI and observed a decrease in the epithelial markers E-cadherin and cytokeratin-18 (CK18) by Western blot as early as 3 days post-treatment (Supplementary Fig. 3C), suggesting that EMT initiation precedes the establishment of AAI-induced senescence. In senescent HK-2 cells, expression of additional epithelial markers such as N-cadherin was further diminished (Supplementary Fig. 3D), while mesenchymal markers, including fibronectin, collagen I, and vimentin, were markedly upregulated (Supplementary Fig. 3E), indicating the development of a mesenchymal phenotype. Morphologically, the loss of membrane-localized N-cadherin, which is essential for TEC cell-cell adhesion, was associated with the appearance of enlarged, spindle-shaped mesenchymal cells that lacked intercellular contacts (Supplementary Fig. 3F). Collectively, these findings suggest that AAI-induced EMT is tightly associated with the development of cellular senescence.

### AAI induces NF-κB activation and promotes SASP secretion in HK-2 cells

**Fig. 6:**
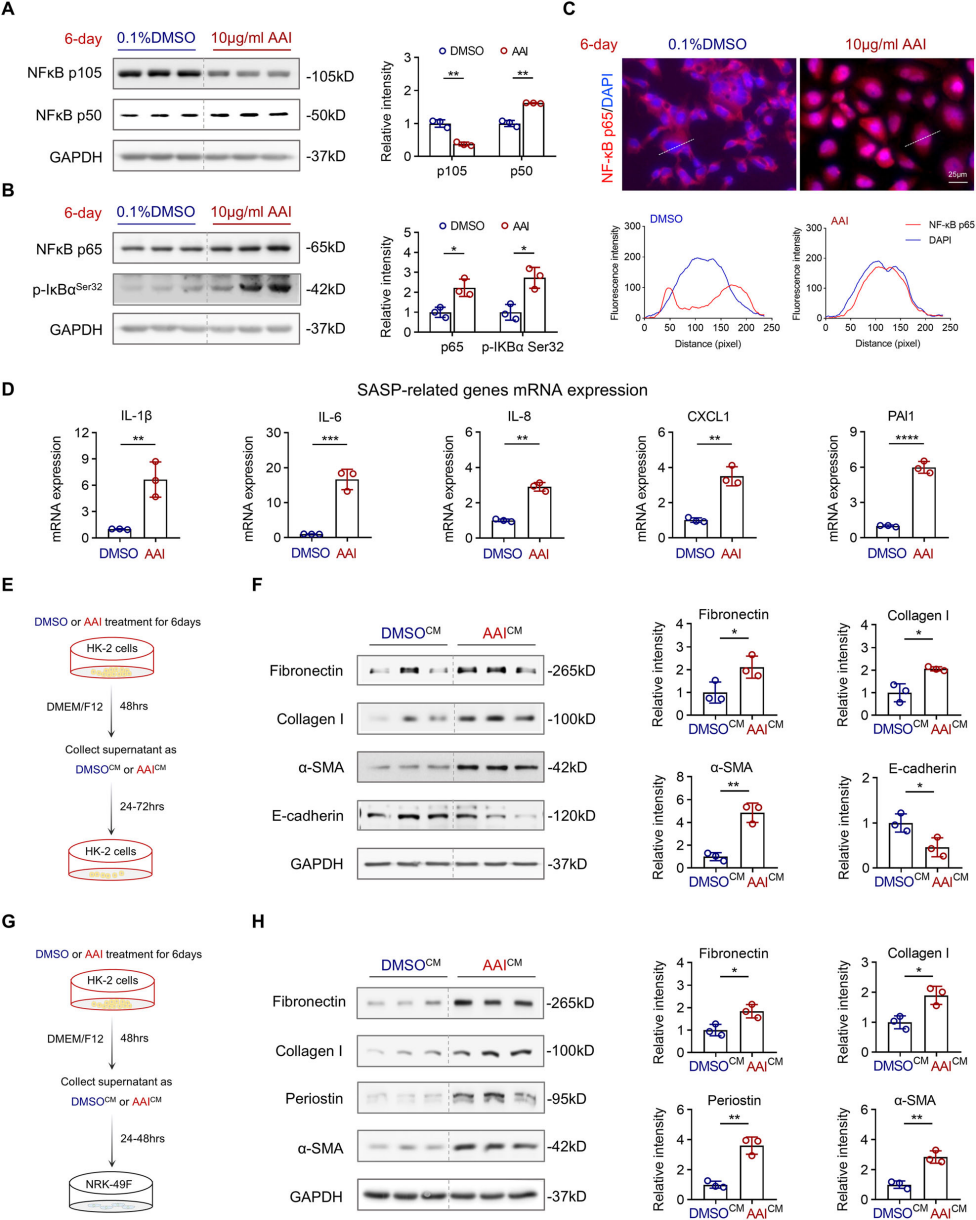
AAI leads to NF-κB activation and SASP production in HK-2 cells, which in turn stimulates EMT and FMT in epithelial cells and fibroblasts, respectively. **A** Representative Western blot analysis and quantification of canonical NF-κB pathway subunit p50 and its precursor p105 of HK-2 cells after 6-day treatment of 10 μg/mL AAI or DMSO. **B** Representative Western blot analysis and quantification of canonical NF-κB pathway subunit p65 and its upstream regulator IκBα of HK-2 cells after 6-day treatment of 10 μg/mL AAI or DMSO. **C** AAI-treated HK-2 cells were subjected to immunofluorescence staining for p65 to show its nuclear translocation, and DAPI was used for counterstaining. The corresponding intensity profiles along the white dotted line (x-axis) show p65 (red) accumulated in the nuclei (blue) of AAI-treated cells (lower panel). **D** qPCR was used to quantify the expression of proinflammatory cytokines (IL-1β, IL-6, and IL-8), chemokines (CXCL1), and profibrotic factors (PAI-1) in 6-day AAI-treated HK-2 cells. **E**, **F** Experimental design of conditional medium (CM) derived from senescent HK-2 cells and treatment with HK-2 for 1–3 days. HK-2 cells were harvested for EMT marker analysis using Western blot. **G**, **H** Experimental design of CM derived from senescent HK-2 cells and treatment with kidney fibroblast (NRK-49F cells) for 1–2 days. NRK-49F were harvested for FMT marker analysis using Western blot. n = 3 per condition, *p < 0.05, **p < 0.01, ***p < 0.001, ****p < 0.0001.

Building on our in vivo findings that AAI activates the NF-κB pathway, we next investigated whether AAI similarly induces NF-κB signaling and SASP expression in AAI-induced senescent HK-2 cells. Following 6 days of AAI treatment, we observed a significant upregulation of the NF-κB subunit p50, accompanied by a marked reduction in its precursor form p105, suggesting enhanced proteolytic processing (Fig. 6A). In parallel, the expression of NF-κB p65 was also elevated (Fig. 6B), with prominent nuclear translocation observed via immunofluorescence staining (Fig. 6C). Consistent with NF-κB pathway activation, we detected increased phosphorylation of IκB-α at serine 32 (Ser32) (Fig. 6B), a key regulatory event that targets IκB-α for proteasomal degradation. This degradation releases NF-κB p65/p50 heterodimers, facilitating their nuclear translocation and subsequent activation of downstream target genes [41]. Furthermore, AAI treatment led to upregulation of multiple SASP-associated transcripts, including the proinflammatory cytokines IL-1β, IL-6, and IL-8, the chemokine CXCL1, and the profibrotic factor PAI-1 (Fig. 6D). The deleterious effects of senescent cells are largely mediated by the SASP, which acts in a paracrine manner to influence neighboring cells and sustain a profibrotic microenvironment [34]. To assess the functional consequences of SASP secretion, we collected conditional medium (CM) from senescent HK-2 cells (AAI^CM^) and from proliferating DMSO-treated controls (DMSO^CM^), and applied them to naïve HK-2 cells (Fig. 6E). Compared to controls, AAI^CM^ significantly increased the expression of EMT markers, including fibronectin, collagen I, and α-SMA, while reducing the epithelial marker E-cadherin (Fig. 6F), indicating that SASP promotes EMT in healthy TEC. Given the profibrotic nature of SASP, we further tested whether senescent TEC could stimulate fibroblast activation and promote their transition into myofibroblasts, a major cell type responsible for ECM production in renal fibrosis [4]. To this end, we treated NRK-49F kidney fibroblasts with AAI^CM^ (Fig. 6G) and found that it markedly induced the expression of myofibroblast markers, including fibronectin, collagen I, periostin, and α-SMA (Fig. 6H). Collectively, these findings demonstrate that AAI-induced senescence in TEC activates the NF-κB pathway and drives SASP secretion, which in turn promotes EMT in epithelial cells and FMT in fibroblasts—two key events contributing to the development of kidney fibrosis.

### Survival of AAI-induced senescent TEC depends on MCL-1 in vitro

**Fig. 7:**
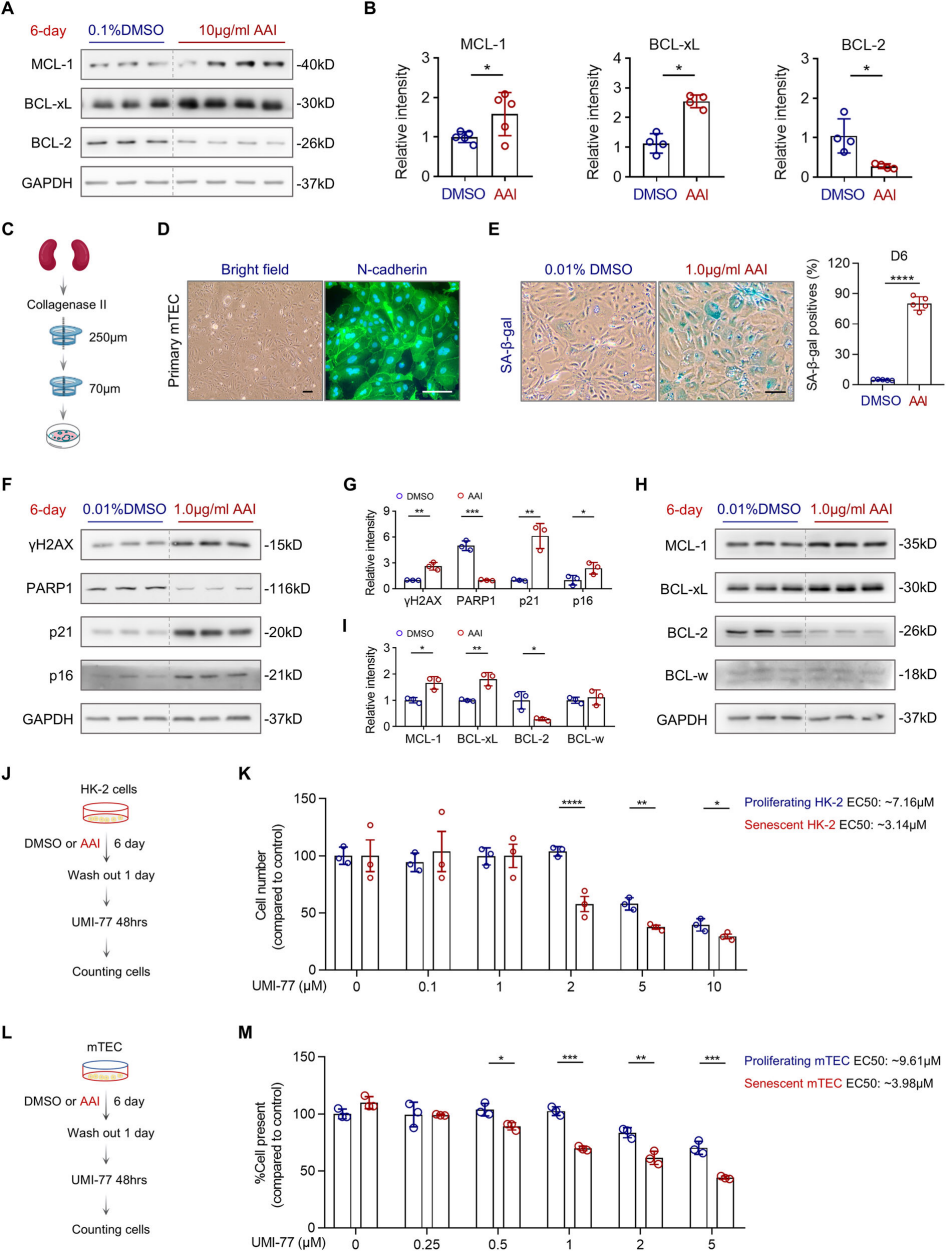
AAI-induced senescent HK-2 cells and primary mTEC exhibit upregulation of anti-apoptotic proteins and enhanced sensitivity to MCL-1 inhibition. **A** Representative Western blot analysis of MCL-1, BCL-xL, and BCL-2 expression in HK-2 cells treated with 0.1% DMSO or 10 μg/mL AAI for 6 days. **B** Quantification of band intensity in (**A**) revealed a significant increase in MCL-1 and BCL-xL, and a decrease in BCL-2 in AAI-treated HK-2 cells compared to controls. **C** Schematic representation of the experimental workflow for the isolation of primary TEC from mouse kidneys (mTEC). **D** N-cadherin, a proximal TEC marker, was used to assess the purity of mTEC. Most cells were positive for N-cadherin staining along the cell membrane. **E** SA-β-gal staining was performed to determine the optimal time point for AAI-induced senescence in mTEC. After 6 days of treatment with 1 μg/mL AAI, there was a significant increase in SA-β-gal^+^ positive cells compared to DMSO-treated controls. **F**, **G** Representative Western blot and quantification of γH2AX, PARP1, p21, and p16 in mTEC treated with 0.01% DMSO or 1.0 μg/mL AAI for 6 days. **H**, **I** Representative Western blot and quantification of anti-apoptotic BCL-2 family proteins (MCL-1, BCL-xL, BCL-2, and BCL-w) in mTEC following 6-day treatment with 0.01% DMSO or 1.0 μg/mL AAI. **J** Schematic of the experimental workflow for UMI-77 treatment. HK-2 cells were exposed to DMSO or AAI for 6 days to induce senescence, followed by a 1-day washout and then treated with the MCL-1 inhibitor UMI-77 for 48 h. **K** Cell viability assessment by trypan blue exclusion assay following UMI-77 treatment revealed that senescent HK-2 cells exhibited increased sensitivity to UMI-77 compared to proliferating cells. **L** Schematic of the experimental workflow for UMI-77 treatment. After a 6-day induction of senescence with AAI or DMSO, mTEC underwent a 1-day washout period followed by treatment with the UMI-77 for 48 h. **M** Trypan blue exclusion assay revealed that senescent mTEC was significantly more sensitive to UMI-77 treatment compared to proliferating mTEC. Scale bar: 50μm. n = 3–5 per condition as indicated, *p < 0.05, **p < 0.01, ***p < 0.001, ****p < 0.0001.

To further investigate the survival mechanisms of senescent TEC in vitro, we first assessed the expression of anti-apoptotic BCL-2 family members in AAI-induced senescent HK-2 cells. Among the candidates, MCL-1 and BCL-xL were markedly upregulated. In contrast, BCL-w was undetectable, and BCL-2 expression was significantly reduced (Fig. 7A, B). We extended these analyses to primary mouse TEC (mTEC), isolated from kidney cortex (Fig. 7C), with N-cadherin staining confirming cell identity and purity (Fig. 7D). Exposure to 1.0 μg/mL AAI for 6 days induced robust senescence in mTEC, as indicated by increased SA-β-gal positivity (Fig. 7E) and elevated expression of canonical senescence markers, including γH2AX, p21, and p16, along with decreased expression of PARP1 (Fig. 7F, G). Analysis of BCL-2 family proteins in senescent mTEC revealed a similar pattern to HK-2 cells: both MCL-1 and BCL-xL were upregulated, while BCL-2 was downregulated and BCL-w remained unchanged (Fig. 7H, I). Notably, this in vitro expression pattern partially diverged from that observed in vivo, suggesting that senescent TEC may rely on distinct survival pathways depending on the microenvironment. Nonetheless, the consistent upregulation of MCL-1 across models highlights its potential role in maintaining senescent cell viability. To evaluate the functional relevance of MCL-1, we treated both proliferating and senescent HK-2 (Fig. 7J) and mTEC (Fig. 7L) with UMI-77, a selective MCL-1 inhibitor. UMI-77 induced preferential cell death in senescent cells, with significantly lower EC_50_ values compared to proliferating counterparts (EC_50_ = 3.14 μM and 3.98 μM for senescent HK-2 and mTEC, respectively, versus 7.16 μM and 9.61 μM in proliferating cells; Fig. 7K, M). Collectively, these findings demonstrate that AAI-induced senescent TEC is selectively vulnerable to MCL-1 inhibition, and that targeting MCL-1 is sufficient to trigger senolytic activity in vitro.

### Single-nucleus transcriptomics identifies a distinct senescent TEC population

To gain insight into the distribution of senescent cells and the pro-survival pathways they rely on at a single cellular level, we utilized snRNA-Seq analysis from a previously published dataset [42]. This dataset was derived from whole-kidney digests of 4 pooled mice per library at day 28 post-AAI injection, which closely aligns with our experimental model. As we found that AAI-induced DNA damage was primarily in the TEC, we focused our analysis on the epithelial compartment. Epithelial cells were initially categorized into distal and proximal tubule populations based on the expression of characteristic marker genes-Slc34a1 for proximal tubules and Slc12a3 and/or Slc8a1 for distal tubules—and visualized according to their condition (naïve or AAN) (Supplementary Fig. 4A, left panel). These populations were then further subclustered into six proximal tubule clusters (Clusters 0–5) and two distal tubule clusters (Clusters 6–7). As shown in Supplementary Fig. 4A (right panel), AAI injection led to a marked reduction in the number of proximal TEC, consistent with our histological findings.

To assess cellular senescence, we first gated key cell cycle inhibitors (Cdkn1a/p21, Cdkn2a/p16, and Cdkn2b/p15) and computed a senescence score based on their expression levels. We observed increased expression of these genes in AAN mice (Supplementary Fig. 4B), with a particularly strong enrichment in proximal tubule Cluster 5, which was also positive for the injury marker Havcr1/KIM1 (Supplementary Fig. 4C). This observation is consistent with our immunofluorescence staining for Cdkn1a/p21, which is colocalized with KIM1. In addition to cell cycle inhibitors, we also assessed senescence by examining the expression of classical SASP components (Tgfb1, Serpine1/PAI-1, Ccl2, Ccn2, Il1b, Mmp3, Cxcl1, and Tnf) and upstream NF-κB pathway regulators (Nfkb1 and Rela/p65). These markers were significantly upregulated in AAN mice (Supplementary Fig. 4D), especially within Cluster 5 (Supplementary Fig. 4E). Collectively, these findings confirm that AAI primarily induces senescence in TEC, with Cluster 5 representing the senescent cell population.

**Fig. 8:**
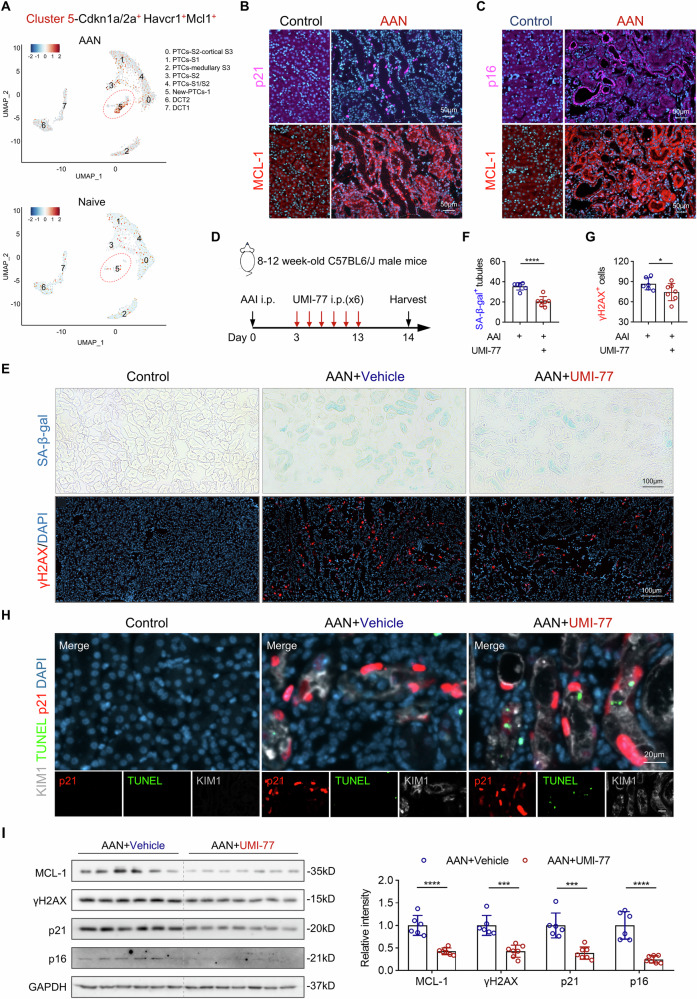
Senolytic MCL-1 inhibitor (UMI-77) initiated in the early phase post-injury ameliorates renal senescence in AAN mice. **A** snRNA-Seq of kidney cells from naive and AAN mice (ArrayExpress database, accession number E-MTAB-9390) was analyzed, identifying pathogenic senescent cells. The UMAP plot displays proximal (clusters 0–5) and distal (clusters 6–7) TEC, colored by the expression levels of Havcr1/KIM1, Cdkn1a/p21, Cdkn2a/p16, and Mcl1 genes. **B**, **C** Representative serial immunofluorescence images showing p21/p16 (Violet) in MCL-1 (Red) expressing renal tubules in AAN mice. White circles indicate similar regions in 2 μm-serial immunostained sections. **D** Experimental schema of senolytic UMI-77 treatment with AAN mice. **E** Representative images of SA-β-gal staining and immunofluorescence staining of γH2AX in aforementioned groups. **F**, **G** Quantification of the number of SA-β-gal^+^ tubules and γH2AX^+^ cells per HPF. **H** Representative images of immunofluorescence staining for p21 and KIM1 co-stained with TUNEL. **I** Representative Western blot analysis and quantification of MCL-1, γH2AX, p21, and p16 of whole kidney lysates. n = 6–7 per condition, *p < 0.05, ***p < 0.001, ****p < 0.0001.

A key feature of senescent cells is their resistance to apoptosis, which is largely attributed to the upregulation of pro-survival BCL-2 family proteins. Consequently, various senolytic strategies targeting these anti-apoptotic proteins have been explored in AKI and CKD models, yielding protective or controversial results. One potential contributor to this variability is the lack of systematic evaluation of pro-survival gene expression in senescent cell populations, particularly in vivo. To address this, we assessed the expression of classical anti-apoptotic genes (Bcl2, Bcl2l1/BCL-xL, Bcl2l2/BCL-w, and Mcl1). We found that these genes were significantly upregulated in AAN kidneys (Supplementary Fig. 4F), with their expression scores enriched in Cluster 5 (Supplementary Fig. 4G), indicating a pro-survival signature within the senescent TEC. Given our therapeutic goal of selectively targeting senescent cells, we next examined the distribution of each anti-apoptotic gene beyond the senescent TEC to identify a candidate with high specificity for senescent TEC and minimal off-target effects. While Bcl2 and Bcl2l1/BCL-xL were highly expressed in senescent TEC (Cluster 15 in Supplementary Fig. 5A, B), they also exhibited high expression in non-senescent cells, potentially leading to undesirable off-target effects. In contrast, Mcl1 and Bcl2l2/BCL-w showed relatively low expression across most cell types but were notably enriched in senescent Cluster 15 (Supplementary Fig. 5A, B). Importantly, de novo synthesis of MCL-1 protein was induced following AAI treatment, whereas BCL-w expression was reduced (Fig. 3A). These findings led us to prioritize MCL-1 as a therapeutic target. Furthermore, Cluster 5 in Fig. 8A showed strong co-enrichment of senescence markers (Cdkn1a/p21, Cdkn2a/p16), the tubular injury marker Havcr1/KIM1, and Mcl1, supporting its identity as a population of senescent, injured TEC. Based on these results, we define this previously uncharacterized cell population as MCL-1⁺ senescent TEC and propose MCL-1 as a promising target for selective senolytic intervention in AAI-induced kidney injury.

### Pharmacological targeting of MCL-1^+^ senescent TEC with UMI-77 mitigates renal fibrosis in AAN mice

**Fig. 9:**
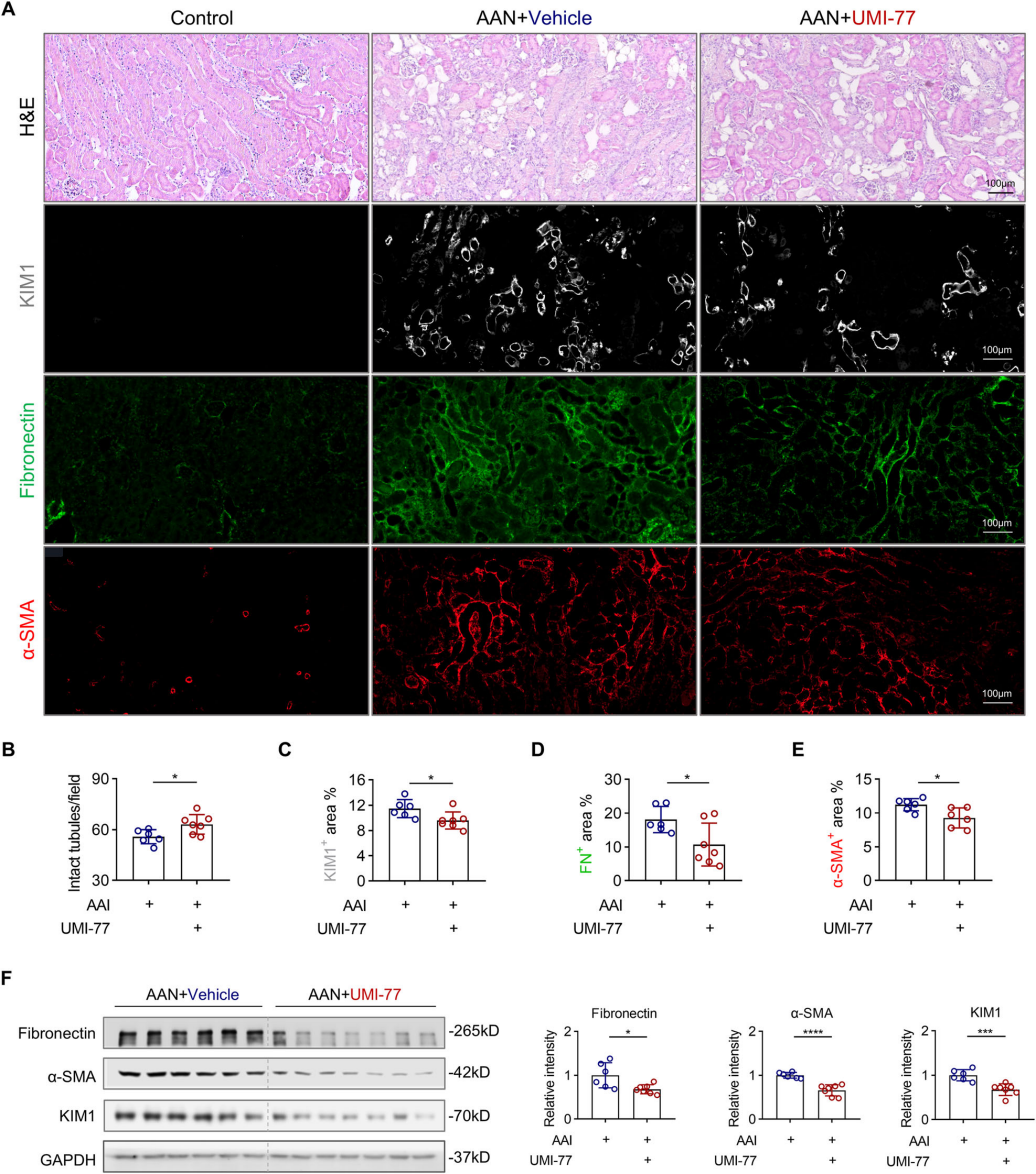
UMI-77 initiated in the early phase post-injury ameliorates kidney fibrosis in AAN mice. **A** Representative image of H&E and immunofluorescence staining of KIM1, fibronectin, and α-SMA in control, AAN+Vehicle, and AAN+UMI-77 mice. **B**–**E** Quantification of the number of intact tubules and percentage of the positive area of KIM1, fibronectin, and α-SMA per HPF. **F** Representative Western blot analysis and quantification of fibronectin, α-SMA, and KIM1 of whole kidney lysates. n = 6–7 per condition, *p < 0.05, ***p < 0.001, ****p < 0.0001.

To substantiate our snRNA-Seq data analysis, we performed immunofluorescence staining for the anti-apoptotic proteins MCL-1, BCL-2, and BCL-xL, and senescent markers p21 and p16 on AAN mice and their respective controls. As expected, MCL-1 colocalized with p21 (Fig. 8B) and p16 (Fig. 8C) in the tubular regions. In contrast, and unexpectedly, BCL-2 and BCL-xL were absent from p21⁺ and p16⁺ tubules (Supplementary Fig. 6), supporting the existence of a distinct MCL-1⁺ senescent TEC under AAN conditions. These findings suggest that selective inhibition of the pro-survival protein MCL-1 may allow for targeted elimination of senescent TEC and potentially slow CKD progression. To address this question, we decided to treat AAN mice with UMI-77. The timing of senolytic treatment influences its efficacy [11]; therefore, in our study, we targeted senescent cells at the acute and chronic phases of AAN injury. For acute-phase targeting, UMI-77 was delivered from day 3 to day 13 post-AAI injection (Fig. 8D). UMI-77-treated mice exhibited decreased SA-β-gal staining and reduced levels of the DNA damage marker γH2AX (Fig. 8E–G), as well as a marked reduction in p21⁺ and p16⁺ TEC (Supplementary Fig. 7), compared to vehicle-treated controls. Notably, UMI-77 induced apoptosis in senescent TEC, as evidenced by the colocalization of KIM1, p21, and TUNEL (Fig. 8H). These observations were corroborated by Western blot analyses, which showed decreased expression of γH2AX, p21, and p16, along with effective suppression of MCL-1 protein expression (Fig. 8I), confirming that UMI-77 effectively inhibited MCL-1 in the kidney. Next, we investigated whether the removal of MCL-1⁺ senescent TEC could attenuate fibrosis development in AAN mice. Histological analysis revealed that acute-phase treatment with UMI-77 conferred a protective effect against AAI-induced tubular injury and subsequent fibrosis (Fig. 9A, B). This was evidenced by an increased number of intact tubules (Fig. 9A, B), accompanied by a marked reduction in KIM1^+^ tubules (Fig. 9A, C). In parallel, interstitial fibrosis was significantly reduced, as demonstrated by decreased staining for fibronectin (Fig. 9A, D) and α-SMA (Fig. 9A, E). These histological findings were further corroborated by consistent changes in protein expression levels observed in Western blot analyses of kidney lysates from the same treatment groups (Fig. 9F).

We further evaluated whether delayed administration of UMI-77 during the chronic stage of injury could confer similar benefits (Supplementary Fig. 8A). Late-phase treatment (days 21–31 post-AAI injection; Supplementary Fig. 8B) had no significant impact on body weight (Supplementary Fig. 8C) but tended to reduce γH2AX⁺ and p21⁺ cell numbers (Supplementary Fig. 8D, E). However, its impact on p16⁺ TEC was limited, showing only a modest, non-significant decline (Supplementary Fig. 8D, E). Correspondingly, late UMI-77 treatment conferred only partial renal protection: while improvements were observed in the number of intact tubules and fibronectin expression, no significant changes were detected in α-SMA or KIM1 levels (Supplementary Fig. 9A–C). Collectively, these results demonstrate that early senolytic targeting of MCL-1⁺ senescent TEC using UMI-77 effectively attenuates tubular injury and interstitial fibrosis in the AAN model, while delayed intervention offers limited therapeutic benefit.

### Senolytic ABT-263 fails to improve and may exacerbate renal fibrosis in AAN mice

Despite our findings indicating that BCL-2 family members other than MCL-1 did not colocalize with senescence markers, we evaluated the effects of ABT-263—a widely used senolytic targeting both BCL-2 and BCL-xL-at various time points. As anticipated, extensive analysis demonstrated that ABT-263 failed to improve senescence or fibrosis markers in both early and chronic phases following AAI administration (Supplementary Figs. S10–S14). Notably, ABT-263 treatment initiated on days 7 or 21 post-AAI aggravated renal fibrosis (Supplementary Figs. 11, 12 and 14), while at day 14 we observed mice dying before our harvest endpoint (Supplementary Fig. 13). Furthermore, despite partially restoring body weight (Supplementary Fig. 14B), ABT-263 treatment unexpectedly increased senescence (Supplementary Fig. 14C–F, I), tubular injury (Supplementary Fig. 14C, H), and fibrosis (Supplementary Fig. 14C, J) by day 35 post-AAI. These findings suggest that senolytics targeting BCL-2 and BCL-xL do not confer renal protection in the AAN model and may, in fact, exacerbate disease progression. Taken together, our data indicate that senescent cells in the AAN model rely on MCL-1, rather than BCL-2 or BCL-xL, for survival. We further confirm that MCL-1⁺ senescent TEC represents a pathological cell population that can be selectively targeted through MCL-1 inhibition to mitigate chronic kidney injury. In contrast, senolytic approaches that target non-MCL-1 pathways may be ineffective or even detrimental in this context.

## Discussion

Senescence is a phenomenon that refers to a state of stable cell cycle arrest often caused by factors like DNA damage [8]. Recent studies have shown that eliminating senescent cells, either genetically or pharmacologically, can help preserve kidney function during aging and CKD [10, 43]. However, the specific role of TEC senescence in the AKI-to-CKD transition remains a subject of debate, largely due to the heterogeneity of senescent cell populations, the timing of senescent cell depletion, and the complexity of their pro-survival mechanisms.

Among renal cell types, TEC is the primary site of senescence in injured kidneys, as observed in both patients [44–46] and experimental models [8, 13, 47, 48]. However, commonly used AKI-to-CKD models, such as IRI and UUO, induce damage across multiple renal cell types [20], making it challenging to isolate the specific contributions of TEC senescence. To overcome this limitation, we utilized the AAN model, which selectively induces DNA damage in TEC [49], allowing us to study their role in the AKI-to-CKD transition while minimizing contributions from senescence in other renal cell types. Indeed, a single injection of AAI not only successfully recapitulated key pathological features of the AKI-to-CKD transition-such as tubular apoptosis-but also robustly induced tubular cell senescence. This senescent phenotype was confirmed through multiple lines of evidence in both in vivo and in vitro settings: i) In vivo, AAI selectively induced DNA damage in KIM1⁺ TEC, while in vitro, it triggered irreversible G2/M cell cycle arrest in HK-2 cells; ii) AAI-treated HK-2 cells exhibited classic senescent morphology, characterized by flattened and enlarged cell shapes, with EMT potentially contributing to this phenotype; iii) TEC exposed to AAI expressed canonical senescence markers, including SA-β-gal, p53, p21, and p16; iv) Senescent TEC upregulated anti-apoptotic proteins-particularly MCL-1-under both in vivo and in vitro conditions, consistent with their enhanced resistance to apoptosis; vi) Finally, AAI treatment led to sustained activation of the NF-κB pathway and upregulation of SASP factors, further supporting the establishment of a senescent state. Together, these findings establish the AAN model as a robust and physiologically relevant platform for studying TEC-specific senescence, offering distinct advantages over traditional AKI models by providing cellular specificity and translational relevance.

Importantly, co-staining of senescence markers (p21, p16) with tubular injury markers (KIM1, NGAL) revealed spatial and temporal differences in senescence pathway activation in AAN. p21 was predominantly localized to injured proximal TEC at early stages, whereas p16 appeared later and extended to both proximal and distal TEC (Fig. 2). A similar temporal pattern was observed in vitro, with early p21 induction followed by delayed p16 upregulation (Fig. 5). These results align with the model in which p21 governs early cell cycle arrest, while p16 maintains long-term senescence [33]. Despite AAI-induced DNA damage being largely restricted to proximal TEC, a senescent phenotype also emerged in distal TEC at later stages, characterized by p16⁺ cells. This suggests that senescent proximal TEC may promote paracrine senescence in neighboring cells (i.e., distal TEC) via SASP, a hypothesis supported by prior reports of senescence propagation through SASP signaling [50]. Thus, the AAN model not only enables the study of distinct senescence pathways in proximal and distal TEC but also serves as a powerful tool for investigating different tubular segments crosstalk and identifying key SASP components responsible for mediating paracrine senescence.

Another important aspect to consider in previous studies [11, 12, 15, 17] is their experimental design, where senolytics were often administered preemptively before the onset of senescence. This raises concerns that the observed protective effects may not solely stem from the elimination of senescent cells but rather from broader drug-induced effects on key signaling pathways. Consequently, this approach may have led to an overestimation of the contribution of senescence to kidney injury. To address this, we first defined the temporal dynamics of senescence in AAN. Senescent TEC emerged between days 3 and 7 post-injury, as evidenced by p21 upregulation and SA-β-gal positivity (Fig. 2), and persisted for at least 35 days (Supplementary Fig. 14). Based on this timeline, we administered UMI-77 either early or late during senescence development. Strikingly, only early intervention effectively reduced tubular injury and fibrosis (Figs. 8 and 9), while late treatment showed limited benefit (Supplementary Figs. 8 and 9). The differential efficacy of UMI-77 at early versus late disease stages remains unclear. We hypothesize that, in early AAN, only TEC undergoes senescence, allowing for their selective elimination. However, at later stages, SASP-mediated signaling may extend senescence to other renal cells, such as fibroblasts and endothelial cells. Indeed, even within the same organ, the impact of senescence on fibrosis appears to be cell-type dependent. For instance, in the liver, senescent hepatic stellate cells can limit fibrosis by suppressing collagen synthesis [51], while hepatocyte senescence exacerbates liver fibrosis through TGF-β-mediated signaling [52]. Given that kidney fibroblasts share functional similarities with hepatic stellate cells, it is plausible that senolytic treatment at later disease stages may also eliminate beneficial senescent fibroblasts, undermining therapeutic efficacy. These findings underscore the importance of targeting senescent cell populations at the appropriate stage and in a cell-type-specific manner.

To explore the impact of survival pathway specificity, we evaluated ABT-263, a senolytic targeting BCL-2 and BCL-xL [53]. While ABT-263 has demonstrated protective effects in models such as reversible UUO [12], unilateral IRI [12], and multiple low-dose cisplatin-induced CKD [10], we sought to evaluate its efficacy in our AAN model. Although ABT-263 efficiently eliminated AAI-induced senescent HK-2 cells in vitro (data not shown), it failed to confer protection in vivo. In fact, certain dosing conditions resulted in worsened outcomes, including increased mortality. This discrepancy may be attributed to differences in injury severity across models. In reversible UUO and uIRI, the presence of a compensatory contralateral kidney likely mitigates ABT-263’s potential off-target toxicity, thus allowing its protective effects to manifest. Similarly, the cisplatin-induced CKD model involves relatively mild injury, potentially explaining the observed therapeutic benefits in that context. These findings suggest that ABT-263 may provide benefits in settings of mild or unilateral kidney injury but may be harmful in more severe, bilateral damage as seen in AAN. Moreover, ABT-263 showed limited specificity in our model: snRNA-seq analysis revealed that BCL-2 and BCL-xL are broadly expressed across various renal cell types, with minimal differential expression between AAN and naïve mice (Supplementary Fig. 5). Furthermore, immunofluorescence data showed that BCL-2 and BCL-xL do not colocalize with p21⁺ or p16⁺ TEC (Supplementary Fig. 6), suggesting limited specificity for senescent cells in AAN mice.

In contrast, MCL-1 expression was selectively upregulated in a subset of senescent TEC, as shown by single-cell clustering and colocalization with p21, p16, and KIM1 (Fig. 8). Additional in vitro experiments confirmed that senescent HK-2 and mTEC exhibit greater sensitivity to the MCL-1 inhibitor UMI-77 at lower concentrations compared to proliferating cells (Fig. 7), indicating a functional dependency on MCL-1 for survival in the senescent state. This supports a senolytic mechanism of action for MCL-1 inhibition. Notably, this dependency on MCL-1 has also been demonstrated in oncology, where single-cell RNA-sequencing revealed that therapy-induced senescent tumor cells expressed high MCL-1 levels across both BCL-2⁺ and BCL-2^−^ clusters, suggesting that MCL-1 functions as an alternative or complementary pro-survival pathway in senescent cells regardless of BCL-2 status. In in vivo models, MCL-1 inhibition led to complete clearance of senescent tumor cells and metastases, whereas BCL-2 inhibition with Navitoclax resulted in only partial effects [54]. These findings strengthen the rationale for selectively targeting MCL-1 in senescence-associated pathologies. Moreover, they underscore the complexity of senescent cell pro-survival mechanisms across different types of kidney injury.

In summary, our findings demonstrate that MCL-1 is a critical survival factor in senescent TEC during AAI-induced kidney injury and that its selective inhibition with UMI-77 provides therapeutic benefit, particularly when administered early. In contrast, broader senolytics like ABT-263 lack cell-type specificity and may be ineffective or harmful in severe injury contexts. These results underscore the need for precision in senolytic design-taking into account the injury model, treatment timing, and survival pathway dependencies of senescent cell subtypes. Future studies should validate the efficacy of MCL-1-targeted strategies in other models of renal injury and further dissect the diverse roles of senescence across kidney cell populations in fibrosis and CKD progression.

## Methods

### Ethical approval

In vivo experiments were carried out according to the Canadian Council on Animal Care guidelines for the use of laboratory animals, under the supervision and approval of our local animal care committee (Comite de protection des animaux du Centre Integre Universitaire de Sante et de Services Sociaux (CIUSSS) de l’Est-de-l’ile-de-Montreal) with the approved protocol number 2022-2895.

### AAN mice model

Male C57BL/6 mice aged 8–12 weeks were purchased from The Jackson Laboratory. AAN was induced by a single intraperitoneal injection of AAI (5 mg/kg body weight/Bw, Sigma, A5512) as previously described [55]. AAI was dissolved in DMSO at a concentration of 10 mg/mL and stored at −20 °C as a stock solution. The AAI stock solution was further diluted with sterile PBS to a working concentration of 1 mg/mL prior to injection. For example, for a 30 g mouse, 15 μl of the AAI stock solution was diluted in 150 μL of sterile PBS to achieve the final injection solution. The normal control mice were administered the same amount of DMSO. Mice were euthanized under isoflurane anesthesia, and kidneys were collected. A slice of the kidney was fixed in 10% formalin for 24-48 h, then embedded in paraffin for histological and immunofluorescence staining. Kidneys were flash-frozen in liquid nitrogen for quantitative polymerase chain reaction (qPCR) and Western blot analysis.

### UMI-77 senolytic treatment

UMI-77 was prepared in 2% DMSO, 30% PEG 400, and 68% ddH_2_O for in vivo use. AAN mice were intraperitoneally injected with UMI-77 at the dose of 10 mg/kg body weight every other day, as previously described [56, 57], starting at 3 or 21 days after AAI injection and sacrificed at indicated time points.

### Senescence-associated β-galactosidase (SA-β-gal) assay

For tissue SA-β-gal assay, kidney samples were fixed in 4% PFA overnight and incubated in 30% sucrose in PBS as previously described [47]. Frozen sections (8 μm) were air dried for 20 min at room temperature and then incubated at 37 °C for ~2–4 h in fresh prepared SA-β-Gal staining solution (pH = 6) using Senescence β-Galactosidase Staining Kit (Cell Signaling, Cat. No 9860). Tissues were imaged by bright-field microscopy, and positive (blue) tubules were counted manually per HPF. For in vitro SA-β-gal staining, after senescence induction, HK-2 cells or mTEC were washed with PBS and fixed with 4%PFA for 5 min at room temperature. After the removal of the fixative buffer, cells were incubated for ~8 h at 37 °C in fresh staining solution (pH = 6). For quantification analysis, a minimum of 3 images were taken from each well of the 12-well plate using the EVOS XL Core Imaging System. Positive (blue) cells were scored as a percentage of the total cell number.

### Induction of senescence in HK-2 cells

To induce senescence in HK-2 cells, we used AAI, a compound known to cause DNA damage. A 10 mg/mL stock of AAI was created by dissolving it in DMSO. We tested different concentrations of AAI (1, 2, 5, 10, 20 μg/mL) to determine the appropriate incubation conditions. A concentration of 20 μg/mL resulted in increased cell death, while a concentration of ≤5 μg/mL was not enough to trigger a persistent cell cycle arrest. After selecting the optimal concentration of AAI, we used the SA-β-gal assay to identify when senescent cells were present. We successfully induced senescence in HK-2 cells by treating them with 10 μg/mL of AAI for 6 days. The medium was replaced and refreshed every 3 days. As a control, we cultured non-senescent HK-2 cells with only DMSO for an identical period of time.

### HK-2 cells treatment with UMI-77

HK-2 cells were treated with 10 μg/mL AAI for 6 days to induce cellular senescence. DMSO-treated HK-2 cells served as proliferating controls. Following a 24-h recovery period in normal culture medium, both proliferating and senescent HK-2 cells were treated with varying concentrations of UMI-77 (0, 0.1, 1, 2, 5, and 10 μM) for 48 h. Cell viability was subsequently assessed using trypan blue exclusion assay. The half-maximal effective concentration (EC₅₀) values reflecting the senolytic efficacy of each compound were calculated based on linear interpolation of dose-response data.

### Generation of conditioned media (CM)

HK-2 cells were treated with 10 μg/mL AAI for 6 days to induce senescence. On day 6, the cells were washed with PBS 3 times and then incubated in a serum-free medium for 48 h. The CM were collected in a centrifuge tube, and the cells remaining on the dish were counted to normalize CM volumes for cell number. The CM was cleared by brief centrifugation and filtered using 0.22 μm filters, and diluted with serum-free medium to a concentration equivalent to 5 × 10^4^ cells/mL. For CM experiments, 8 × 10^4^ cells were seeded on 6-well tissue culture plates and incubated for 1–3 days in CM supplemented with 10% FBS or 5%FCS for HK-2 cells and NRK-49F, respectively. CM was replenished every 24 h. Cells were harvested for protein extraction.

### Data statistics and analysis

The data were expressed as mean ± standard deviation (SD). Statistical significance was assessed by a one or two-tailed Student’s t-test for two-group comparisons or a one-way ANOVA for multigroup comparisons, followed by a Tukey post hoc test for subgroup comparisons. p < 0.05 was considered significant. Statistical analysis and graphical representation were performed using GraphPad Prism Version 8.0.1.

## Supplementary information

Supplementary Figures

Uncropped images for WB

## Data Availability

All data are available in the main text or the Supplementary Materials. Single-nucleus RNA-sequencing of AAN and naive mice that support the findings of this study are openly accessible with the following accession code no. E-MTAB-9390 in the ArrayExpress database.
