# Supplementary material for: Inhibition of MCL-1 to eliminate senescent cells and mitigate renal fibrosis in aristolochic acid nephropathy
Source: Cell Death Dis. 2025 Nov 26;17(1):56. doi: 10.1038/s41419-025-08268-7 (PMC12824375; doi:10.1038/s41419-025-08268-7)
Supplement: Supplementary file 1 — Supplementary Figures [file 41419_2025_8268_MOESM1_ESM.docx]

**Inhibition of MCL-1 to eliminate senescent cells and mitigate renal fibrosis in aristolochic acid nephropathy**

Peng Gao^1,2^, Schrodinger Cenatus^2,3^, Nathalie Henley^2^, Vincent Pichette^1,2,4,5^, Frédérick A. Mallette^2,3,4^, Jonatan Barrera-Chimal^2^, Casimiro Gerarduzzi^1,2,4,5^*

^1^Department of Pharmacology and Physiology, Faculty of Medicine, University of Montreal, Montreal, Quebec, Canada

^2^Maisonneuve-Rosemont Hospital Research Center, Center affiliated with the University of Montreal, Montreal, Quebec, Canada

^3^Department of Biochemistry and Molecular Medicine, Faculty of Medicine, University of Montreal, Montreal, Quebec, Canada

^4^Department of Medicine, Faculty of Medicine, University of Montreal, Montreal, Quebec, Canada

^5^Division of Nephrology, Maisonneuve-Rosemont Hospital, Montreal, Quebec, Canada

**RUNNING TITLE:** MCL-1 inhibition mitigates renal fibrosis

**List of Supplementary materials:**

Supplementary material and methods;

Supplementary Figure S1-Figure S14;

Supplementary Table S1-Table S3;

Supplementary references.

**SUPPLEMENTARY MATERIALS AND METHODS**

**Chemicals and reagents**

Dimethylsulfoxide (DMSO), ammonium persulfate (APS), Ponceau S, and tetramethylethylenediamine (TEMED) were purchased from Sigma-Aldrich (Oakville, ON, Canada). Acrylamide, ethylenediaminetetraacetic acid (EDTA), bis-acrylamide, sodium dodecyl sulfate (SDS), Tween 20, methanol, Tris-base, and glycine were purchased from VWR International (Mississauga, ON, Canada), and 10% buffered formalin and 4% Paraformaldehyde (PFA) was purchased from Fisher Scientific (Saint-Laurent, QC, Canada). UMI-77 (HY-18628) and ABT-263 (Navitoclax, HY-10087) were purchased from MedChemExpress (MCE, South Brunswick, NJ, USA). Ethanol (P016EAAN, Greenfield Global, ON, Canada), polyethylene glycol 400 (PEG400, 8.07485, Oakville, ON, Canada), phosal 50 propylene glycol (PG, Lipoid GmbH, 368315, Ludwigshafen Germany). All chemicals were of ACS grade or higher.

**ABT-263 senolytic treatment**

ABT-263 was prepared in 10% ethanol, 30% PEG 400, and 60% Phosal 50 PG. ABT-263 was administered by gavage (50 mg/kg body weight) daily or every other day as indicated. The dose selection was based on a previous study^50^. AAN mice were treated with ABT-263 or vehicle at 3, 7, 14, and 21 days after AAI injection. After each indicated time point, mice were sacrificed for intensive analysis.

**Histological staining**

Kidney tissue was fixed in 10% formalin. Paraffin-embedded sections at 5-μm thick were used. Sections were stained with hematoxylin and eosin (H&E) following the manufacturer's protocol. The number of intact tubules was determined manually with the help of the cell colony counter plugin in ImageJ (National Institutes of Health). For fibrosis evaluation, sections were deparaffinized and rehydrated before being stained in Picro Sirius Red (PSR) solution for 1 hour. Mix Direct Red 80 (365548, Sigma, Oakville, ON, Canada) with the saturated aqueous solution of picric acid (A38-212, Fisher Scientific, Saint-Laurent, QC, Canada) to make 0.1% PSR staining solution. A total of 6 independent fields in kidneys from each mouse were analyzed and the mean value was plotted.

**Immunofluorescence staining**

Kidney sections were deparaffinized and rehydrated. Epitope retrieval was performed using citrate buffer (10 mM sodium citrate and 0.05% Tween 20, pH 6.0) at 95 °C for 10~20 min. After blocking with 5% donkey serum, the sections were incubated with the respective antibodies at the dilutions specified in Supplemental Tables S1 and S2. Fluoroshield with DAPI (Millipore-Sigma) was used for nuclear staining and mounting. Tile scan images were acquired with a Zeiss AxioObserver.Z1 inverted microscope at 10 × magnification. Nonoverlapping high power fields (HPF) (magnification 400x) were used for quantitative analysis, and the number of positive cells/tubules/mean fluorescence intensity (MFI) was determined as the average of positive signals of at least 6 fields per kidney section. For immunocytochemistry staining, HK-2 cells were fixed with 4% PFA, blocked with 5%BSA, and incubated with p65 (1:50; Santa Cruz Biotechnology, sc-372) at 4°C overnight. After washing with PBS, they were incubated with secondary antibodies conjugated with Alexa Fluor 647. Cells were counterstained with DAPI to visualize the nuclei and examined by aforementioned microscopy.

**Terminal deoxynucleotidyl transferase dUTP nick-end labeling (TUNEL) assays**

TUNEL assays were performed using the In Situ Cell Death Detection Kit, TMR Red (REF 12156792910, Roche) according to the manufacturer’s instructions. Briefly, 5-μm paraffin-embedded kidney sections were deparaffinized and rehydrated. The sections were then permeabilized with a freshly prepared buffer containing 0.1% Triton X-100 and 0.1% sodium citrate for 8 minutes at room temperature, followed by incubation with the TUNEL reagent mixture for 60 minutes at 37°C. After incubation, the sections were washed three times with PBS (5 minutes per wash) and mounted with an antifade mounting medium. Tile scan images were acquired using a Zeiss AxioObserver.Z1 inverted microscope at 10× magnification. TUNEL-positive cells were counted at 400× magnification in six fields per kidney and recorded.

**Western blot analysis**

Cold radioimmunoprecipitation assay (RIPA) buffer (Thermo Fisher Scientific, Waltham, MA) containing phosphatase and protease inhibitors (Roche Diagnostics) was used to homogenize kidney tissues and cell cultures. The concentration of cellular lysate was determined using a bicinchoninic acid (BCA) protein assay kit (Pierce, Cat. No. 23225). Protein lysates (20μg for cellular lysate and 40μg for kidney lysate) for separation by electrophoresis on 8~12% polyacrylamide gels containing 0.4% SDS, followed by transfer onto 0.22 μm nitrocellulose membranes (Amersham Protran, GE Healthcare Life science, Mississauga, Canada) or polyvinylidene difluoride membranes (Bio-Rad, Mississauga, Canada). The membranes were incubated with the primary and secondary antibodies at the dilutions specified in Supplemental Tables S1 and S2. Bands were detected with the Clarity Max Western ECL Substrate from Bio-Rad Laboratories (Hercules, USA). Results were analyzed using ImageJ.

**qPCR**

RNA was extracted from HK-2 cells or murine kidneys using TRIzol (Invitrogen, Burlington, ON, Canada), and then purified using the RNeasy Mini kit (Qiagen, Toronto, ON, Canada) according to the manufacturer’s protocol. One microgram of total RNA was reverse transcribed into cDNA using SuperScript VILO cDNA Synthesis kit with ezDNase (Invitrogen). qPCR was performed using SsoAdvanced Universal SYBR Green Supermix (Bio-Rad) on an ABI 7500 Real-Time PCR System (Applied Biosystems, Foster City, CA). The list of mouse-specific primers can be found in Supplemental Table S3. All samples were measured with technical triplicates and normalized against average housing-keeping gene (HK) expression (glyceraldehyde-3-phosphate dehydrogenase (GAPDH), hypoxanthineguanine phosphoribosyltransferase (HPRT1), ribosomal protein lateral stalk subunit P0 (*Rplp0*)). Primer amplification efficiencies were calculated for each gene and changes in the mRNA expression were determined using the Pfaffl method.

**Single-nucleus mRNA sequencing (snRNA-Seq) data analysis**

Transcriptomes of single cells from naïve and AAN mouse kidneys from the ArrayExpress database (E-MTAB-9390) were analyzed^1^. The CellRanger count pipeline aligns sequencing reads in FASTQ files to a mouse reference transcriptome and performs cell calling, generating barcode-gene matrices for each sample. The barcode-gene matrix was analyzed using the R package Seurat. In Seurat, cells for individual samples were retained if they contained ≥400 genes and genes identified in three or more nuclei. The integrated dataset was then scaled and processed with principal component analysis (PCA), FindNeighbors, and FindClusters (resolution = 0.64, to achieve the same number of clusters as in the reference, 27 clusters in total). Final clustering results were visualized using Uniform Manifold Approximation and Projection (UMAP). Differential gene expression (DGE) analysis was performed to obtain the gene markers of each cluster versus all other cells (significance was defined as a gene with an adjusted p-value = 0.05, a ≥ 0.25 average log-fold difference between the two groups of cells, and presence detected in at least 10% of cells in either of the two populations). We used canonical markers of kidney cell populations to identify major cell types in the kidney: podocyte (Nphs1), endothelial cells (Flt1), mesangial cells (Igfbp5), juxtaglomerular (JG) cells (Ren1), PTCs (Slc34a1), proliferative cells (Top2a and Mki67), descending thin limb (Aqp1), ascending thin limb (Clcnka), thick ascending limb (TAL; Slc12a1 and Umod), distal convoluted tubule 1 (Slc12a3), and distal convoluted tubule 2 (Slc12a3 and Slc8a1), connecting tubule (Slc8a1), principal cell (PC)-outer medullary collecting duct and inner medullary collecting duct (Aqp2), intercalated cells type A (Atp6v1b1 and Slc4a1) and type B (Atp6v1b1 and Slc26a4), transitional epithelium (Upk1b), immune cells (Ptprc), and fibroblasts (Pdgfrb and Cfh). Senescence-related genes include Cdkn1a, Cdkn2a, and Cdkn2b. Senescence-associated secretory phenotype (SASP)-related genes include Tgfb1, Serpine1 (Pai1), Ccl2 (Mcp1), Cxcl1, Tnf, Il1b, Mmp3, and Ccn2, and SASP upstream regulator-NF-κB pathway-related genes include NF-κB1 and RelA (p65). Anti-apoptotic-related genes include Bcl2, Bcl2l1 (Bcl-xL), Bcl2l2 (Bcl-w), and Mcl1.

**Cell culture**

The human proximal tubule cell line (HK-2, derived from male normal kidney) and the rat kidney fibroblast (NRK-49F, derived from normal rat kidney) were obtained from American Type Culture Collection (ATCC, USA) and maintained in a humidified atmosphere of 5% CO_2_ at 37°C with Dulbecco’s Modified Eagle’s Medium/F12 (DMEM/F12) supplemented with heat-inactivated 10% fetal bovine serum (FBS) (Gibco/ Life Technologies, USA) or DMEM supplemented with heat-inactivated 5% fetal calf serum (FCS), respectively.

**Cell cycle analysis**

DMSO- or AAI-treated HK-2 cells were trypsinized and prepared single cell suspension at ~1 x 10^6 cells/mL in PBS buffer. Aliquot 200µl cells (~2 x 10^5) in a 1.5 ml Eppendorf tube and add 500µl cold 100% ethanol dropwise and gently mix by pipetting in and out at least ten times (ethanol final concentration will be ~70%) and fixed at 4°C for 1hr. After being washed with PBS, the cells were incubated out of light in PBS containing 50 μg/mL propidium iodide (PI, VWR, item No. 89139-066, Mississauga, ON, Canada) and 10μg/mL RNaseA (Sigma, R4875, Oakville, ON, Canada) at 4°C for 2.5hrs. Then, before measurement, the cell samples were resuspended in PBS and analyzed with a FACS Calibur flow cytometry (Becton Dickinson, USA).

**Cell viability assay**

The cell viability was evaluated using trypan blue. For the trypan blue exclusion assay, the culture medium was aspirated and reserved. After trypsinization and centrifuge, cells were resuspended in complete medium and mixed with 0.4% trypan blue, and the number of viable cells was counted using a hemacytometer (Bright-line, Hausser Scientific, PA, USA).

**Isolation and culture of primary mouse kidney tubular epithelial cells (mTEC)**

mTEC was isolated and cultured under sterile conditions from collagenase II-digested cortical fragments of kidneys harvested from ~4 to 8 weeks old mice, following a modified version of previously described protocols^2^. Renal cortices were manually minced into ~1 mm³ fragments in ice-cold PBS and subsequently digested in collagenase II solution (2 mg/mL in PBS) at 37°C for ~30 minutes, with gentle pipetting every 10 minutes to facilitate dissociation. Digestion was halted by adding an equal volume of stop buffer (5% FBS in PBS) and mixing thoroughly. The resulting cell suspension was sequentially filtered through 250 μm and 70 μm sieves. Larger proximal tubule (PT) fragments retained on the 70 μm sieve were collected by reverse flushing with warm PBS, followed by centrifugation at 300 × g for 5 minutes. The pellet was washed with PBS and resuspended in primary mTEC culture medium, consisting of DMEM/F12 supplemented with 5% heat-inactivated FBS, 15 mM HEPES, 4 mg/L dexamethasone, 0.7% ITS Premix (Corning, Cat. No. 354351), 0.25 mM sodium pyruvate, 0.05 mM L-ascorbic-2-phosphate, 100 IU/mL penicillin, and 100 μg/mL streptomycin. The isolated PT fragments were maintained in culture without agitation for 48 hours at 37°C in a humidified incubator (95% air, 5% CO₂). The culture medium was first replaced at 48 hours and subsequently changed every two days. By ~day 7, cells had formed a confluent monolayer, designated as Passage 0. mTEC were characterized by the expression of proximal epithelial markers N-cadherin^3, 4^, with negative staining for the mesenchymal marker α-SMA. Only passage 1 mTEC were used for in vitro experiments.

**Induction of senescence in mTEC**

Senescence in mTEC was induced using the same strategy as that applied to HK-2 cells. Specifically, mTEC were treated with 1.0 μg/mL AAI for 6 days, with the culture medium refreshed every 3 days. As a control, non-senescent mTEC were cultured in parallel with 0.01% DMSO for the same duration.

**mTEC treatment with UMI-77**

mTEC were treated with 1.0 μg/mL AAI for 6 days to induce cellular senescence. DMSO-treated mTEC served as proliferating controls. Following a 24-hour recovery period in normal culture medium, both proliferating and senescent mTEC were treated with varying concentrations of UMI-77 (0, 0.25, 0.5, 1, 2, and 5μM) for 48 hours. Cell viability was subsequently assessed using trypan blue exclusion assay. The half-maximal effective concentration (EC_50_) values reflecting the senolytic efficacy of each compound were calculated based on linear interpolation of dose-response data.

**
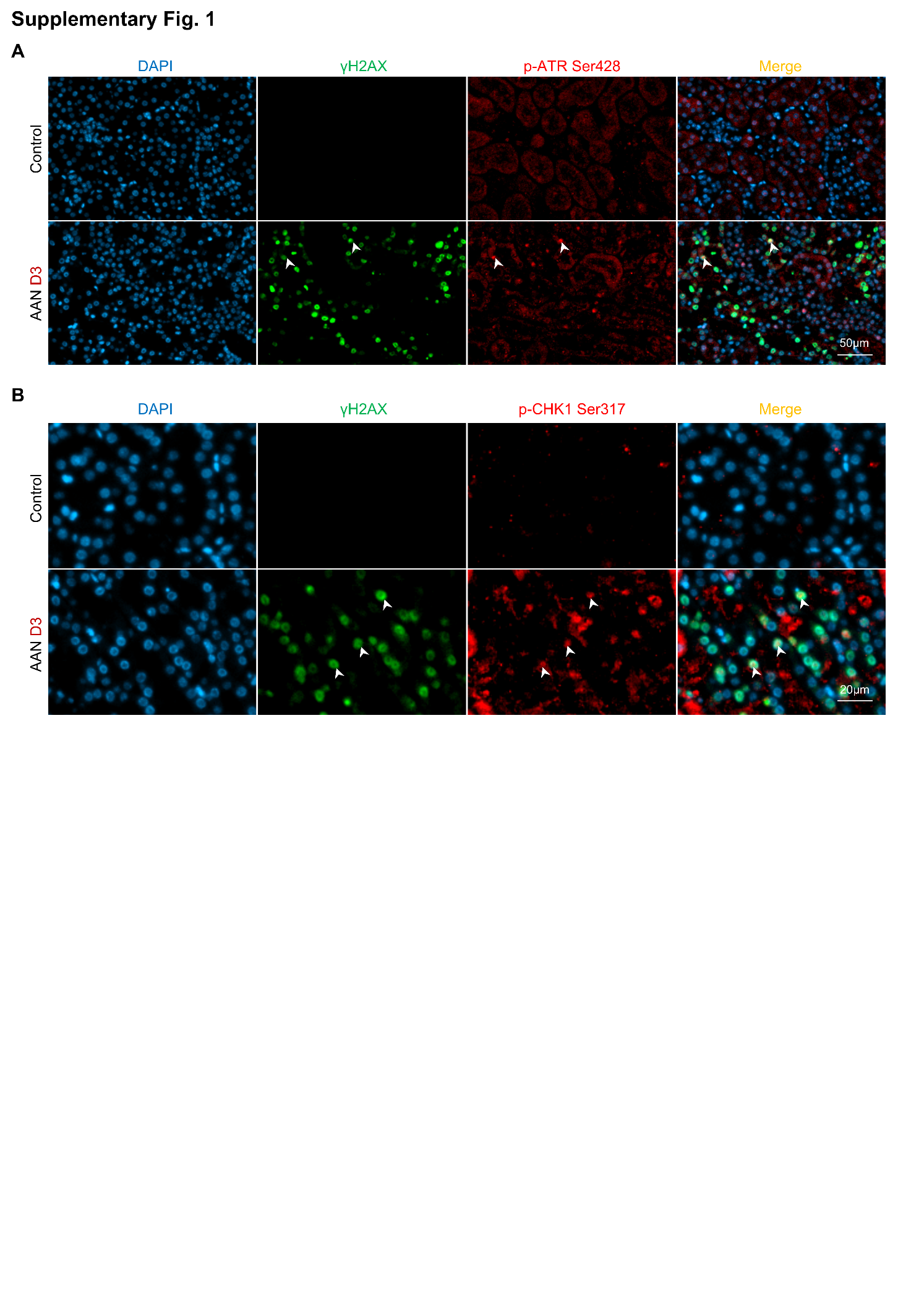
**

**Supplementary Fig. 1 p-ATR ser 428 and p-CHK1 Ser 317 expression are increased in AAN kidney at day 3 with a weaker γH2AX co-localization. A** Representative images of γH2AX co-stained with p-ATR Ser 428 at day 3 after AAI injection. **B** Representative images of γH2AX co-stained with p-CHK1 Ser 317 at day 3 after AAI injection. n = 6 for each group. n = 6 for each group.


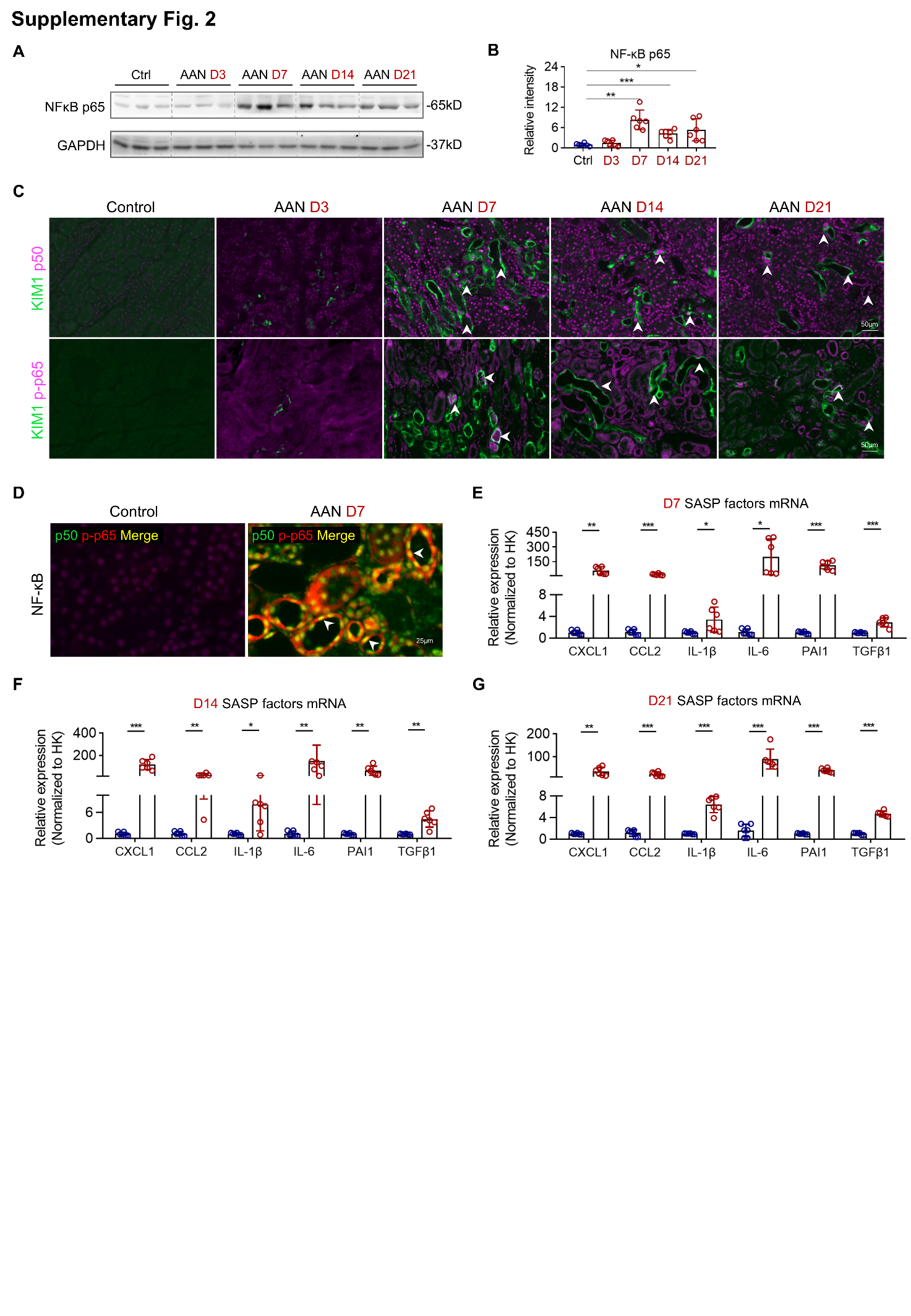


**Supplementary Fig. 2 AAI induces NF-κB activation and SASP production in the kidney of AAN mice. A**, **B** Representative Western blot analysis and quantification of the p65 subunit of canonical NF-κB pathways in whole kidney lysates. **C** Representative images of KIM1 co-stained with phospho-p65 (p-p65) and p50 subunit at various time points after AAI injection. **D** Representative image of p50 and p-p65 nuclear co-staining at day 7 post-AAI injection. **E**-**G** qPCR analysis of representative SASP factors of whole kidney lysates harvested at D7, 14, and 21 post-AAI injection. n = 6 for each time point, p*<0.05, p**<0.01, p***<0.001.


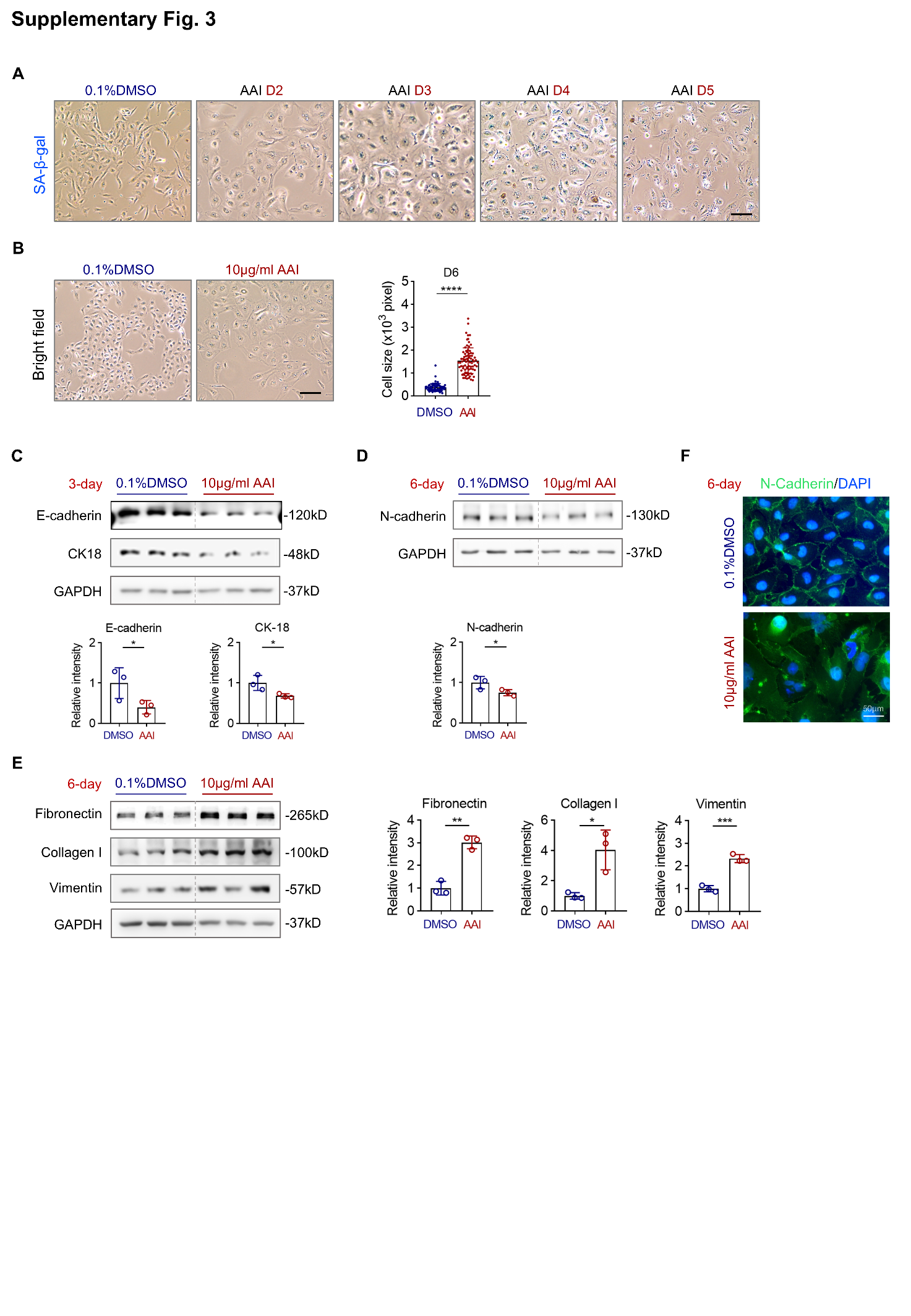


**Supplementary Fig. 3 AAI induces senescence and an early mesenchymal phenotype in human tubular epithelial cells *in vitro*. A** HK-2 cells were treated with 10μg/mL AAI for 1~5 days and SA-β-gal assay was performed to screen the occurrence of senescence over time. **B** Representative images of cellular morphology of DMSO- and AAI-treated HK-2 cells (6-day treatment) and quantification of cellular size. **C**, **D** HK-2 cells were treated with 10μg/mL AAI or DMSO for the indicated time and Western blot was used to analyze the expression of epithelial markers (E-cadherin, CK-18, and N-cadherin) and quantification of the above markers. **E** Representative Western blot analysis and quantification of mesenchymal markers (fibronectin, collagen I, and vimentin) of HK-2 cells after 6-day treatment of 10μg/mL AAI or DMSO. **F** Immunofluorescence staining of N-cadherin in HK-2 cells treated with DMSO or AAI for 6 days. n = 3 per condition, p*<0.05, p**<0.01, p***<0.001, p****<0.0001.


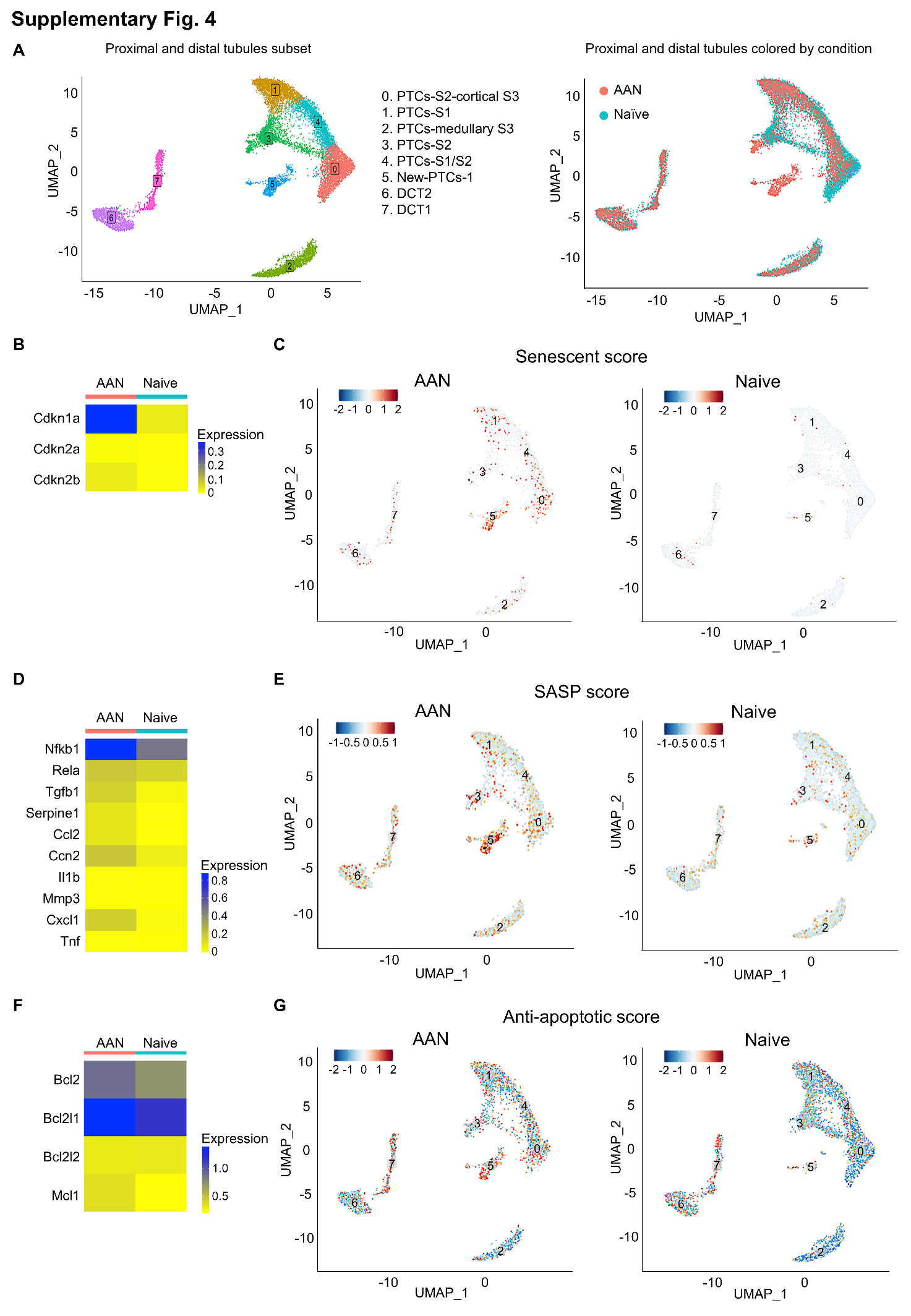


**Supplementary Fig. 4 Tubular cell senescence profile in AAN using snRNA-seq analysis.** **A** UMAP annotation of cell clusters among the population of proximal (0~5) and distal (6~7) TEC. **B** Heatmap of differentially expressed senescence-related genes between AAN and naïve kidneys. **C** UMAP plot of proximal and distal TEC colored by senescence module score. **D** Heatmap of differentially expressed SASP-related genes between AAN and naïve kidneys. **E** UMAP plot of proximal and distal TEC colored by SASP module score. **F** Heatmap of differentially expressed anti-apoptotic genes between AAN and naïve kidneys. **G** UMAP plot of proximal and distal TEC colored by anti-apoptotic module score.

**
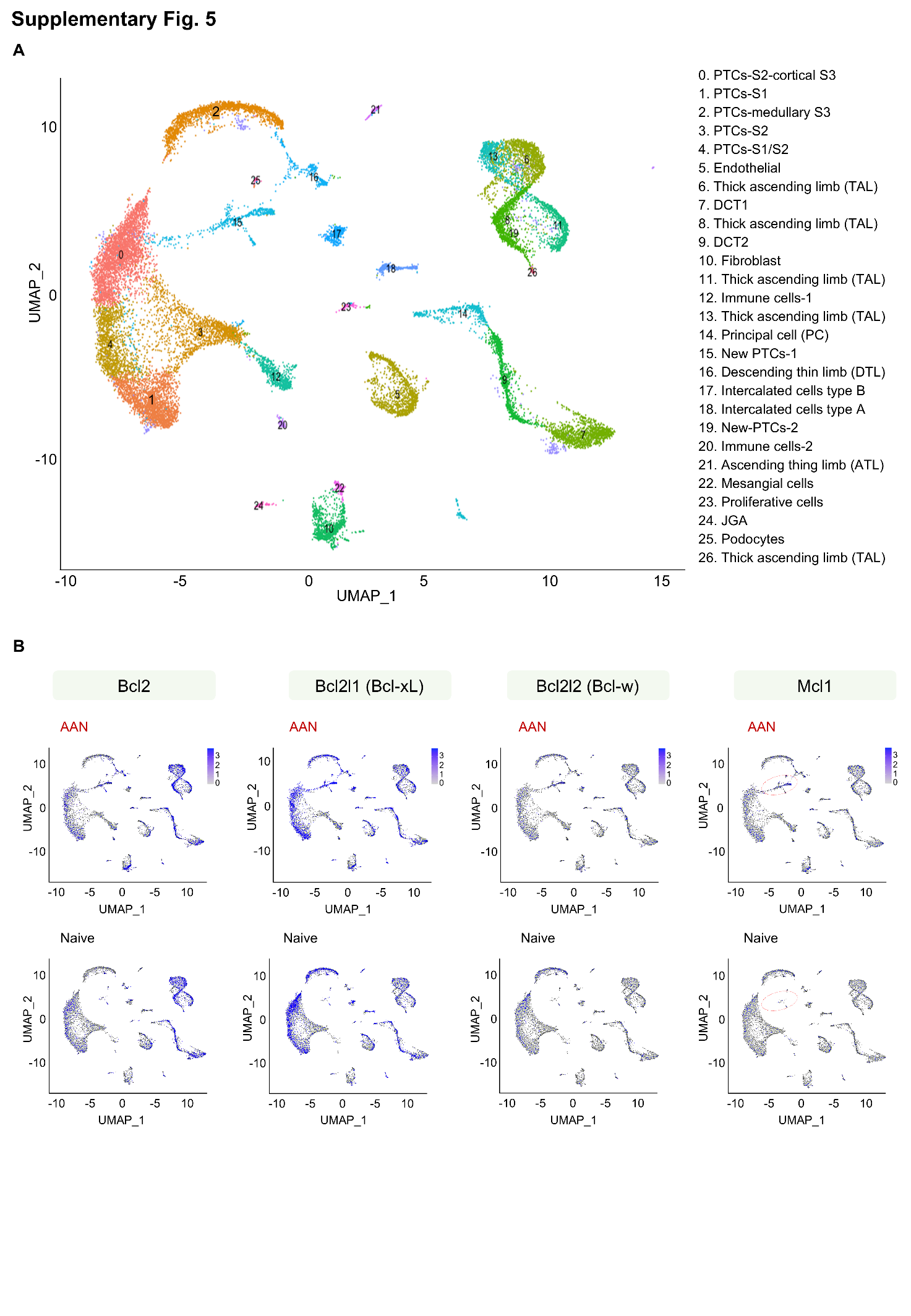
**

**Supplementary Fig. 5 Tubular cell senescence profile in AAN using snRNA-seq analysis.** **A** UMAP annotation of all kidney cell clusters found in AAN and naïve samples. **B** All kidney cells from AAN and naive mice colored by individual anti-apoptotic genes (Bcl2, Bcl2l1/BCL-xL, Bcl2l2/BCL-w, and Mcl1).


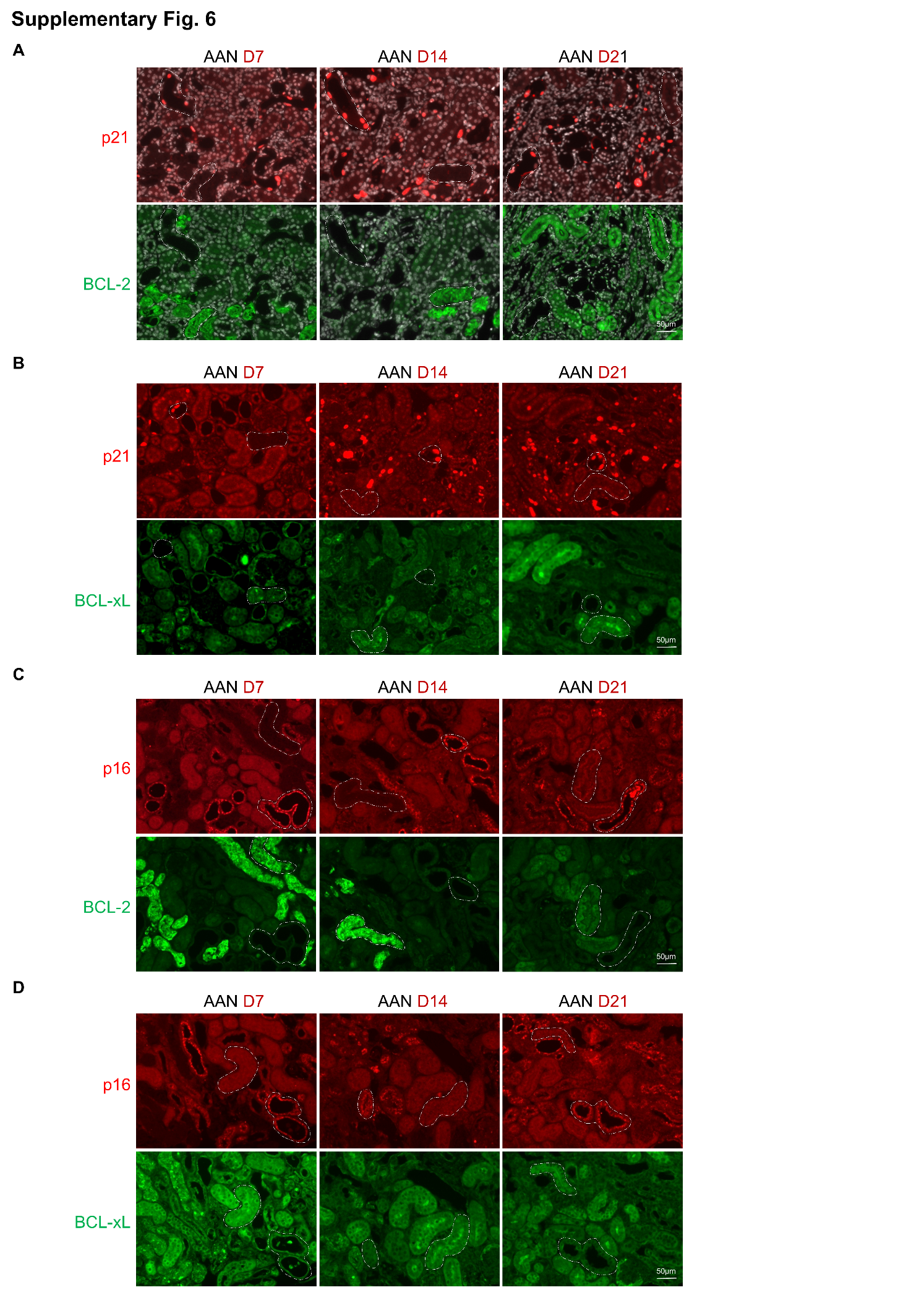


**Supplementary Fig. 6 The anti-apoptotic proteins BCL-2 and BCL-xL (targets of ABT-263) do not colocalize with p21^+^ or p16^+^ renal tubules in AAN mice.** **A**, **B** Representative serial immunofluorescence images showing p21 (Red) in BCL-2 (Green) or BCL-xL (Green) expressing renal tubules in AAN mice. White circles indicate similar regions in 2μm serial immunostained sections. **C**, **D** Representative serial immunofluorescence images showing p16 (Red) in BCL-2 (Green) or BCL-xL (Green) expressing renal tubules in AAN mice. White circles indicate similar regions in serial immunostained sections.


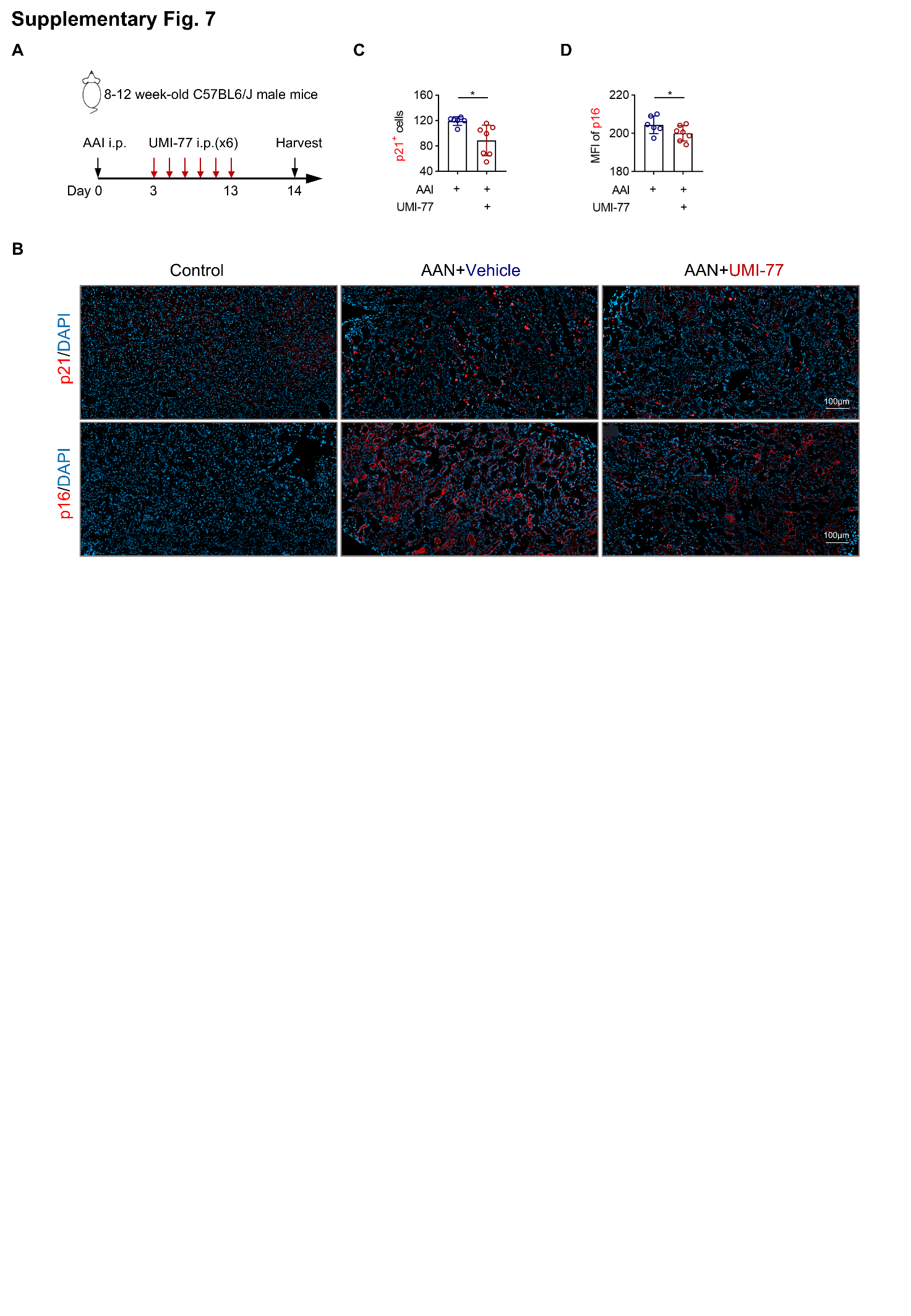


**Supplementary Fig. 7 Senolytic UMI-77 initiated in the early phase post-injury decreases senescence-related markers in AAN mice.** **A** Experimental schema of senolytic UMI-77 treatment with AAN mice. **B** Representative images of immunofluorescence staining of senescent markers (p21 and p16) in aforementioned groups. **C**, **D** Quantification of the number of p21 positive cells or MFI of p16 positive area per HPF. n=6-7/group, p*<0.05.


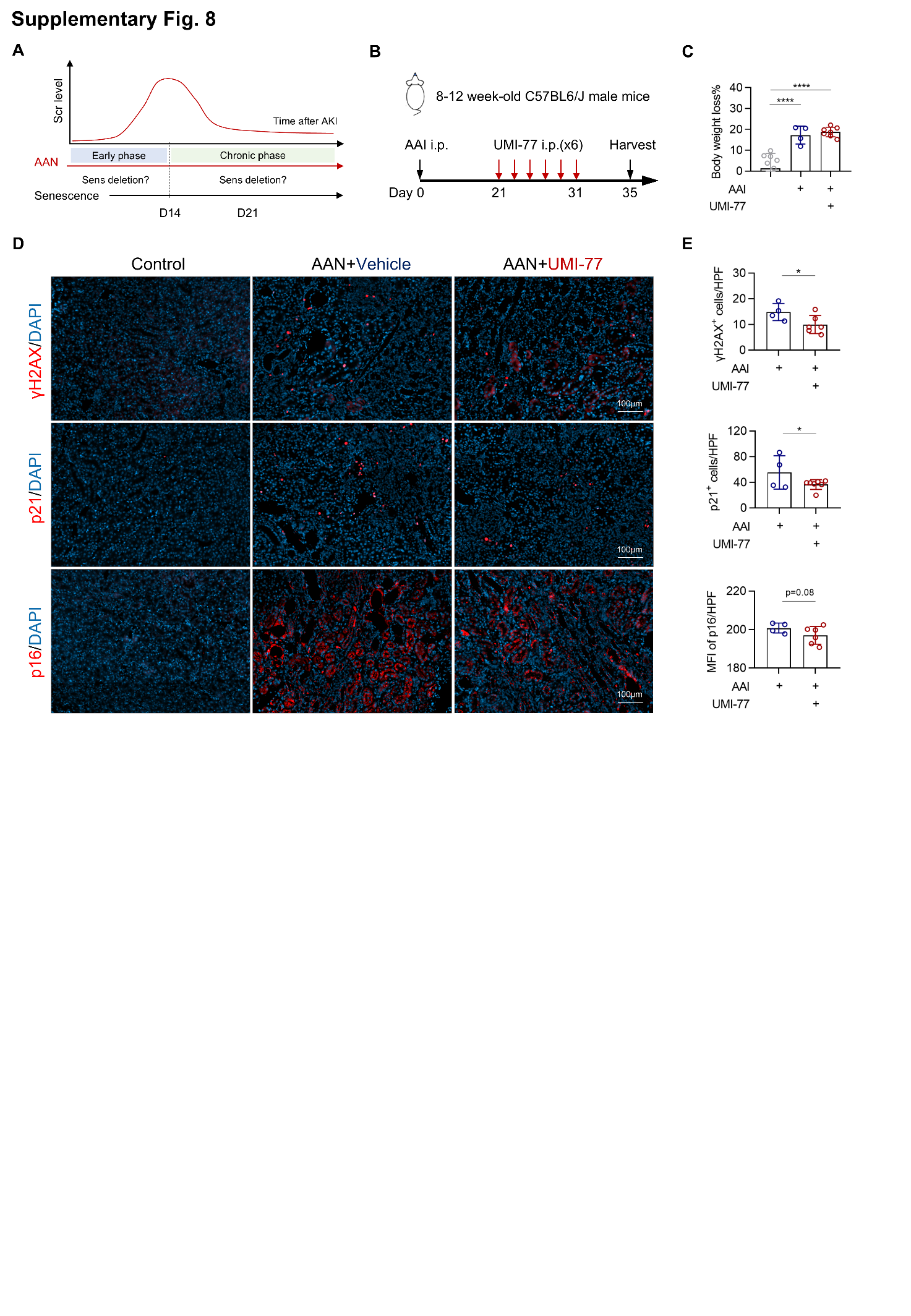


**Supplementary Fig. 8** **UMI-77 initiated in the late phase post-injury partially decreases renal senescence in AAN mice.** **A** Timeline of acute and chronic phase in AAN model defined by the level of serum creatinine (Scr). **B** Experimental schema of senolytic UMI-77 treatment with AAN mice. Senescent cells (Sens) were deleted using UMI-77 in each phase. **C** Changes of body weight loss in control, AAN+Vehicle, and AAN+UMI-77 groups. **D** Representative images of immunofluorescence staining of senescent markers (γH2AX, p21 and p16) in aforementioned groups. **E** Quantification of the number of γH2AX, p21 positive cells or MFI of p16 positive area per HPF. n = 4~7 per condition, p*<0.05, p****<0.0001.


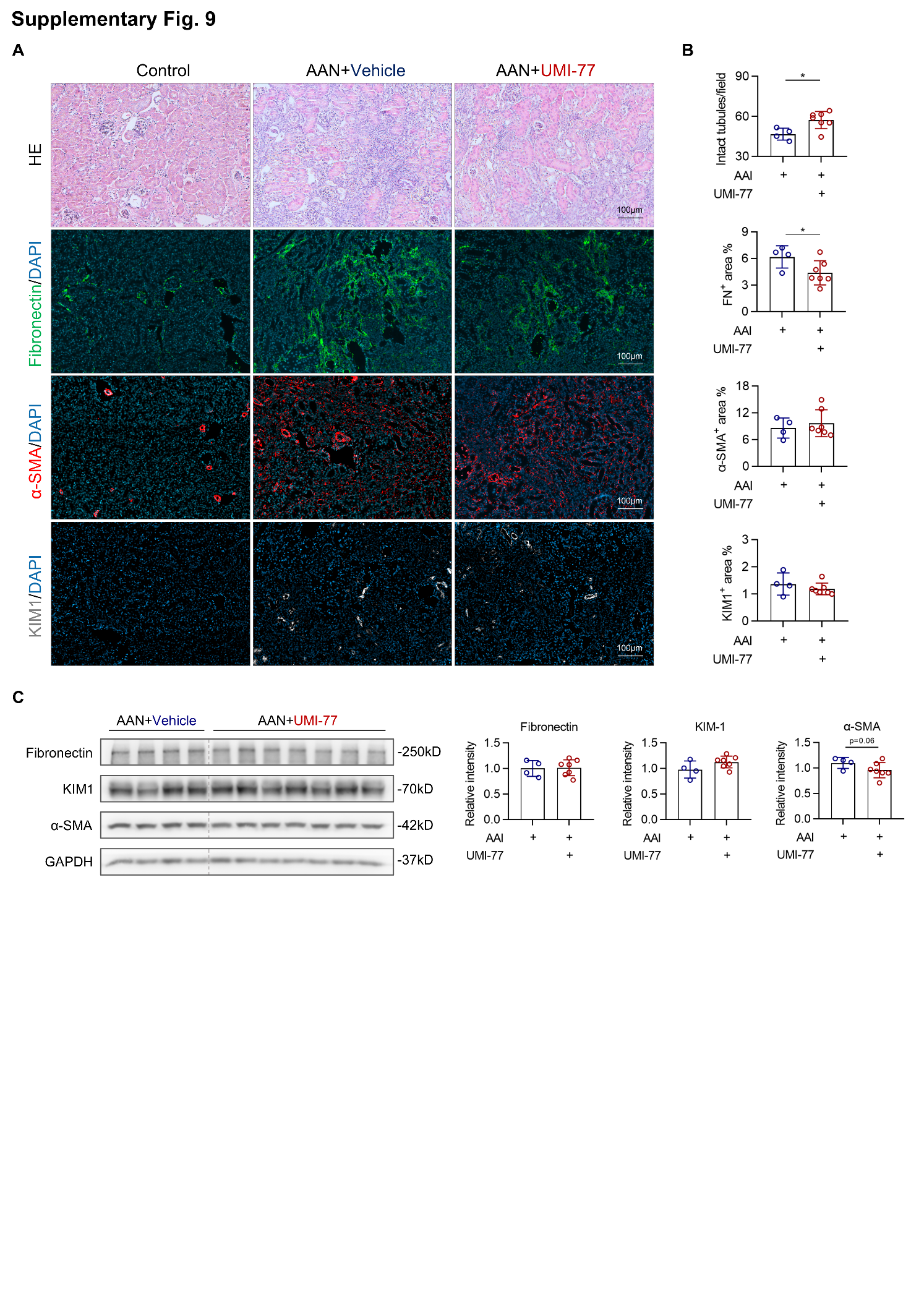


**Supplementary Fig.** **9 Senolytic UMI-77 administrated in the late phase post-injury partially ameliorates kidney fibrosis in AAN mice.** **A** Representative image of histological staining (H&E) and immunofluorescence staining of fibrotic markers (fibronectin and α-SMA) and tubular injury marker (KIM1) in control, AAN+Vehicle, and AAN+UMI-77 groups. **B** Quantification of the number of intact tubules and percentage of the positive area of FN, α-SMA and KIM1 per HPF. **C** Representative Western blot analysis and quantification of fibronectin, α-SMA, and KIM1 of whole kidney lysates. n = 4~7 per condition, p*<0.05, p***<0.001, p****<0.0001.


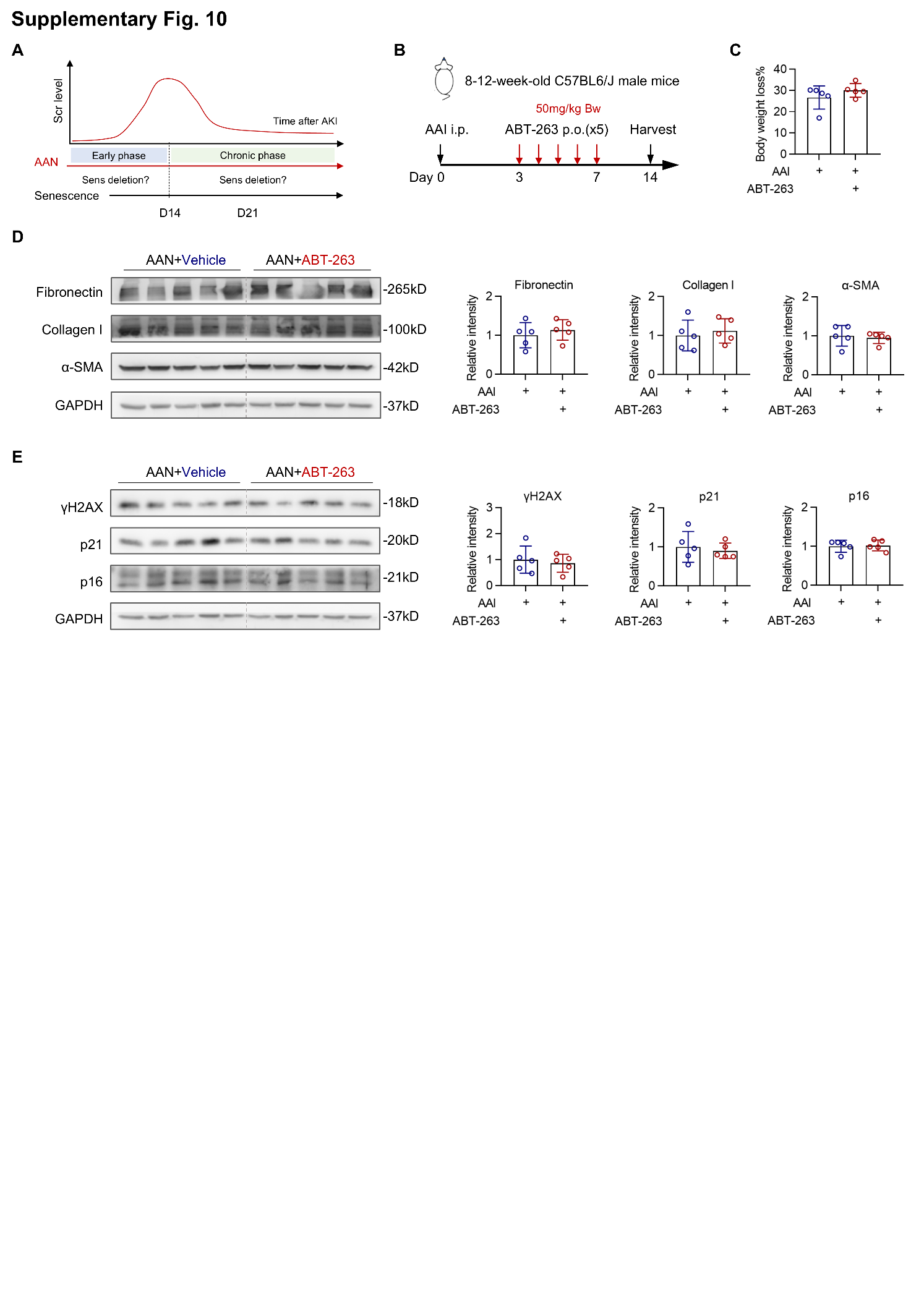


**Supplementary Fig. 10** **Senolytic ABT-263 initiated in the early acute phase post-injury has no effect on renal fibrosis and senescence in AAN mice. A** The disease course of AAN and can be generally divided into 2 phases based on the change of Scr level: acute and chronic phase. Senescent cells (Sens) were deleted using senolytics (ABT-263) in each phase. **B** Experimental schema of senolytic ABT-263, an inhibitor of BCL-2 and BCL-xL, treatment with AAN mice. **C** Changes of body weight loss in AAN+Vehicle and AAN+ABT-263 groups. **D** Representative Western blot analysis and quantification of fibrotic markers (fibronectin, collagen I, and α-SMA) of whole kidney lysates. **E** Representative Western blot analysis and quantification of senescent markers (γH2AX, p21 and p16) of whole kidney lysates. n = 5 per condition.


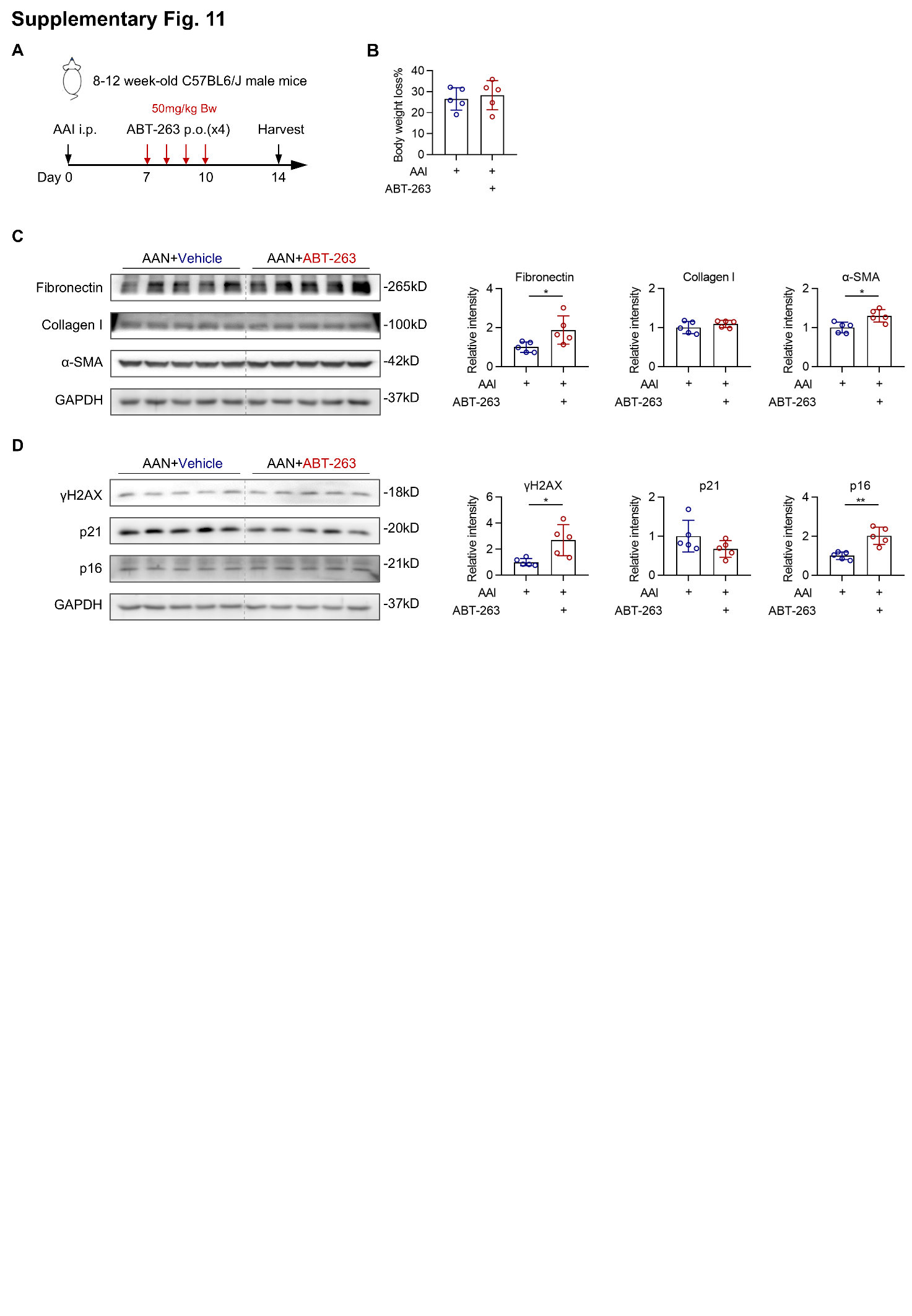


**Supplementary Fig. 11** **ABT-263 daily treatment in the late acute phase post-injury increases the expression of certain kidney senescent and fibrotic markers in AAN mice.** **A** Experimental schema of senolytic ABT-263 treatment during the late acute phase of AAN. **B** Changes of body weight loss in AAN+Vehicle and AAN+ABT-263 groups. **C** Representative Western blot analysis and quantification of fibrotic markers (fibronectin, collagen I, and α-SMA) of whole kidney lysates. **D** Representative Western blot analysis and quantification of senescent markers (γH2AX, p21 and p16) of whole kidney lysates. n = 5 per condition, p*<0.05, p**<0.01.


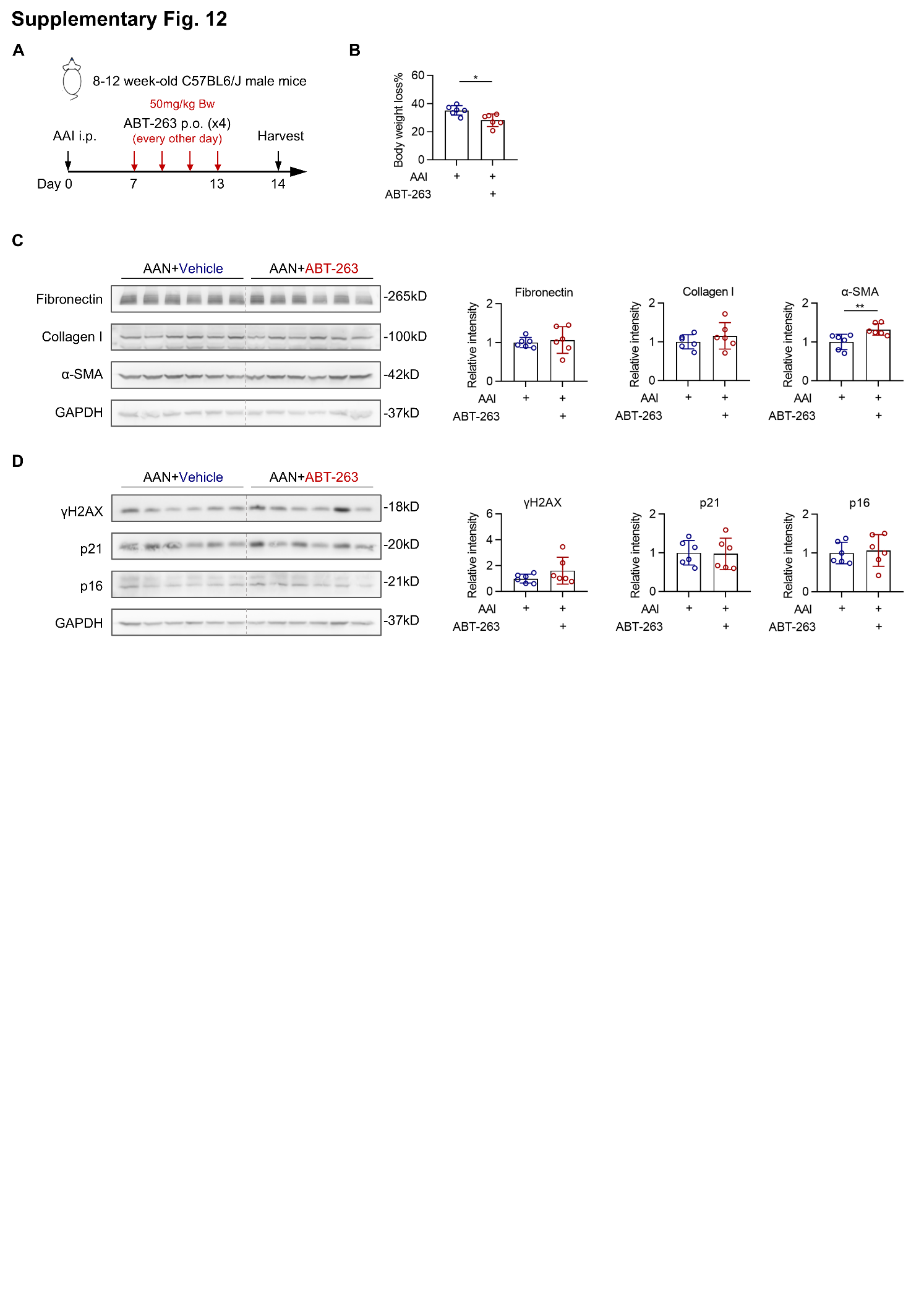


**Supplementary Fig. 12** **ABT-263 intermittent treatment in the late acute phase post-injury tends to increase kidney fibrosis in AAN mice.** **A** Experimental schema of senolytic ABT-263 intermittent treatment during the late acute phase of AAN. **B** Changes of body weight loss in AAN and AAN+ABT-263 groups. **C** Representative Western blot analysis and quantification of fibrotic markers (fibronectin, collagen I, and α-SMA) of whole kidney lysates. **D** Representative Western blot analysis and quantification of senescent markers (γH2AX, p21 and p16) of whole kidney lysates. n = 5 per condition, p*<0.05, p**<0.01.


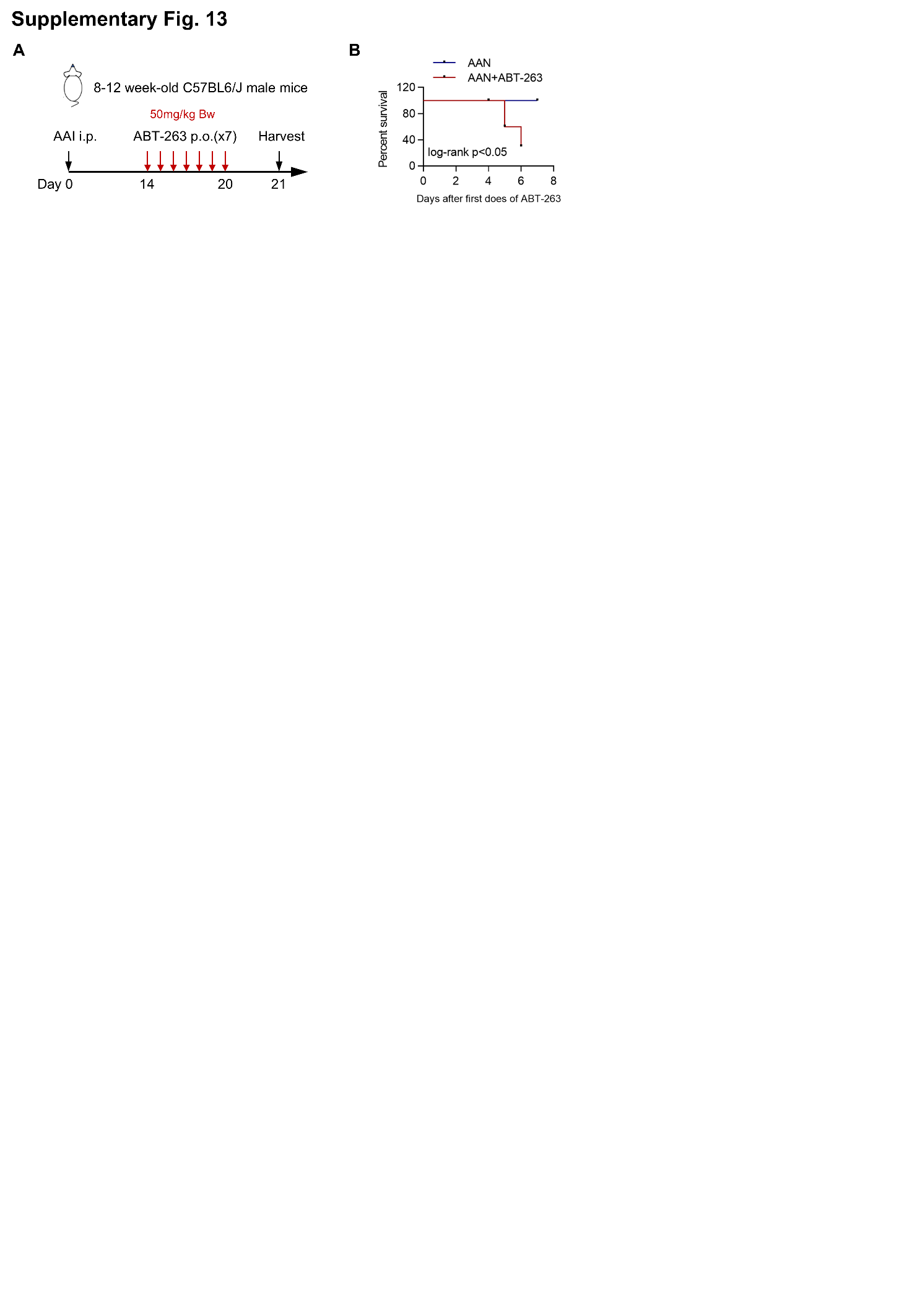


**Supplementary Fig. 13** **ABT-263 given in the recovery phase post-injury is lethal to AAN mice.** **A** Experimental schema of senolytic ABT-263 treatment during the recovery phase of AAN. **B** Survival analysis of ABT-263 and Vehicle-treated AAN mice, n = 6 per condition.


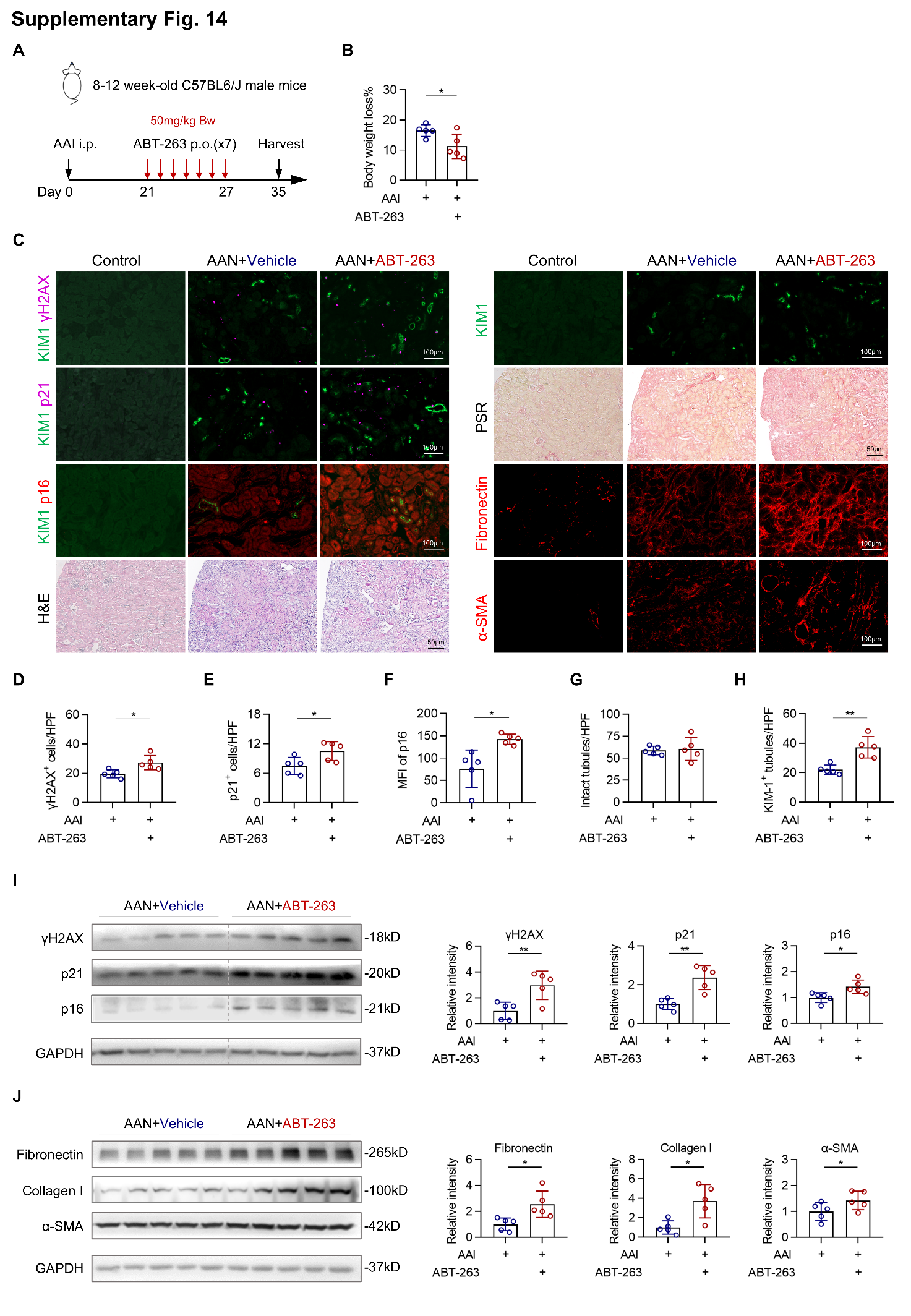


**Supplementary Fig. 14** **ABT-263 initiated in the chronic phase post-injury increases kidney senescence and fibrosis in AAN mice.** **A** Experimental schema of senolytic ABT-263 treatment during the chronic phase of AAN. **B** Changes of body weight loss in AAN+Vehicle and AAN+ABT-263 groups. **C** Representative images of histological staining (H&E and PSR) and immunofluorescence staining of senescent markers (γH2AX, p21, p16), tubular injury marker (KIM1), and fibrotic markers (fibronectin and α-SMA) in control, AAN+Vehicle, and AAN+ABT-263 groups. **D**, **E** Quantification of the number of positive cells of γH2AX, and p21 per HPF. **F** Quantification of MFI of p16 per HPF. **G** Quantification of the number of intact tubules per HPF. **H** Quantification of the number of positive KIM1^+^ tubules per HPF. **I** Representative Western blot analysis and quantification of senescent markers (γH2AX, p21 and p16) of whole kidney lysates. **J** Representative Western blot analysis and quantification of fibrotic markers (fibronectin, collagen I, and α-SMA) of whole kidney lysates. n = 5 per condition, p*<0.05, p**<0.01.

**Table S1: Primary antibodies for Western blot and immunofluorescence**

| Primary Antibodies | Vendor | Cat. number | Applications | | |
| --- | --- | --- | --- | --- | --- |
|  |  |  | WB | IHC-P | ICC |
| γH2AX | CST | 9718 | 1:1000 | 1:250 |  |
| MCL-1 | CST | 5453 | 1:1000 | 1:100 |  |
| BCL-xL | CST | 2764 | 1:1000 | 1:300 |  |
| BCL-w | CST | 2724 | 1:1000 |  |  |
| KIM1 | R&D | AF1817 | 1:500 | 1:125 |  |
| N-cadherin | BD | 610920 | 1:1000 |  | 1:100 |
| NGAL | Santa Cruz | sc515876 |  | 1:100 |  |
| p53 | Santa Cruz | sc126 | 1:500 |  |  |
| NF-κB p65 | Santa Cruz | sc372 | 1:1000 |  | 1:50 |
| NF-κB p-p65 | Santa Cruz | sc136548 |  | 1:100 |  |
| NF-κB p50/p105 | Santa Cruz | sc7178 |  | 1:200 |  |
| p-IκBα | Santa Cruz | sc7977 | 1:500 |  |  |
| Vimentin | Santa Cruz | sc6260 | 1:1000 |  |  |
| Periostin | Santa Cruz | sc398631 | 1:500 |  |  |
| p21 (human) | Abcam | Ab109520 | 1:2000 |  |  |
| p21 (Mouse) | Abcam | Ab188224 | 1:1000 | 1:250 |  |
| GAPDH | Abcam |  | 1:1000 |  |  |
| p16 | Abcam | Ab189034 | 1:500 | 1:100 |  |
| Fibronectin | Abcam | Ab23750 | 1:3000 | 1:400 |  |
| E-cadherin | Abcam | Ab15148 | 1:500 |  |  |
| BCL-2 | Abcam | Ab182858 | 1:2500 | 1:200 |  |
| Collagen I | Proteintech | 14695-1-AP | 1:500 |  |  |
| Bax | Proteintech | 60267-1-Ig | 1:2500 |  |  |
| Cytokeratin 18 | Proteintech | 108301-1-AP | 1:2500 |  |  |
| α-SMA | Sigma | A2547 | 1:5000 (In vivo) | 1:400 |  |
| α-SMA | Sigma | A2547 | 1:1000 (In vitro) |  |  |
| N-cadherin | BioLegend | 844701 | 1:1000 |  |  |
| Rb | BD | 554136 | 1:250 |  |  |
| p-ATM | Santa Cruz | sc47739 | 1:200 | 1:50 |  |
| ATM | CST | 2873 | 1:1000 |  |  |
| p-ATR | CST | 2853 | 1:1000 | 1:200 |  |
| ATR | Santa Cruz | sc515173 | 1:200 |  |  |
| p-CHK1 | CST | 12302 | 1:1000 | 1:250 |  |
| CHK1 | Santa Cruz | sc8408 | 1:200 |  |  |
| p-CHK2 | CST | 2661 | 1:1000 | 1:250 |  |
| CHK2 | CST | 2662 | 1:1000 |  |  |
| PARP1 | CST | 9542 | 1:1000 |  |  |
| γH2AX | Millipore | 05-636 |  | 1:250 |  |
| Cleaved caspase3 | CST | 9661 |  | 1:200 |  |

**Table S2: Secondary antibodies for Western blot and immunofluorescence**

| Secondary Antibodies | Vendor | Cat. number | Applications | | |
| --- | --- | --- | --- | --- | --- |
|  |  |  | WB | IHC-P | ICC |
| Anti-goat Alexa 488 | ThermoFisher | A-11055 |  | 1:300 |  |
| Anti-mouse Alexa Cy3 | Jackson Immunoresearch | 715-165-150 |  | 1:300 | 1:300 |
| Anti-rabbit Alexa 647 | Jackson Immunoresearch | 711-605-152 |  | 1:300 | 1:300 |
| Anti-mouse Alexa 647 | Jackson Immunoresearch | 115-605-003 |  | 1:300 |  |
| Anti-rabbit Alexa Cy3 | Jackson Immunoresearch | 711-165-152 |  | 1:300 |  |
| Anti-rabbit IgG-HRP | Santa Cruz | sc-2357 | 1:2000 |  |  |
| Anti-mouse IgG-HRP | Santa Cruz | sc-516102 | 1:2000 |  |  |
| Anti-goat IgG-HRP | Santa Cruz | sc-2354 | 1:2000 |  |  |

**Table S3: Primers for qPCR**

| Gene | Specie | Forward (5’ to 3’) | Reverse (5’ to 3’) |
| --- | --- | --- | --- |
| Cxcl1 | Mouse | TCACCTCAAGAACATCCAGAGC | AGTGTGGCTATGACTTCGGTTT |
|  | Human | GCCCAAACCGAAGTCATAGCC | ATCCGCCAGCCTCTATCACA |
| Ccl2 | Mouse | CCAATGAGTAGGCTGGAGAGC | GAGCTTGGTGACAAAAACTACAGC |
| IL-1β | Mouse | ATGCCACCTTTTGACAGTGATG | CCAGGTCAAAGGTTTGGAAGC |
|  | Human | CTGTCCTGCGTGTTGAAAGA | TTGGGTAATTTTTGGGATCTACA |
| IL-6 | Mouse | CTCTGGGAAATCGTGGAAATG | AAGTGCATCATCGTTGTTCATACA |
|  | Human | GTGAAAGCAGCAAAGAGGCAC | CAGTGATGATTTTCACCAGGCA |
| IL-8 | Human | AAGGTGCAGTTTTGCCAAGG | GTGTGGTCCACTCTCAATCACT |
| Pai1 | Mouse | TCCTGCCTAAGTTCTCTCTGGA | GGGTTGCACTAAACATGTCAGG |
|  | Human | ATCGAGGTGAACGAGAGTGG | ACTGTTCCTGTGGGGTTGTG |
| Tgfβ1 | Mouse | CCGAAGCGGACTACTATGCTAA | TTCTCATAGATGGCGTTGTTGC |
| Gapdh | Mouse | CGTGGAGTCTACTGGTGTCTTCA | GGCGGAGATGATGACCCTTT |
|  | Human | GTCGGAGTCAACGGATT | AAGCTTCCCGTTCTCAG |
| Hprt1 | Mouse | GGACTGATTATGGACAGGACTGA | ATGTAATCCAGCAGGTCAGCAA |
|  | Human | CCTGGCGTCGTGATTAGTGA | CGAGCAAGACGTTCAGTCCT |
| Rplp0 | Mouse | CCACTTACTGAAAAGGTCAAGGC | TCTGATTCCTCCGACTCTTCCT |
|  | Human | GGAAACTCTGCATTCTCGCTTCCT | CCAGGACTCGTTTGTACCCGTTG |

**Reference**

1. Lu YA, Liao CT, Raybould R, Talabani B, Grigorieva I, Szomolay B*, et al.* Single-Nucleus RNA Sequencing Identifies New Classes of Proximal Tubular Epithelial Cells in Kidney Fibrosis. *Journal of the American Society of Nephrology : JASN* 2021, **32**(10)**:** 2501-2516.

2. Terryn S, Jouret F, Vandenabeele F, Smolders I, Moreels M, Devuyst O*, et al.* A primary culture of mouse proximal tubular cells, established on collagen-coated membranes. *American journal of physiology Renal physiology* 2007, **293**(2)**:** F476-485.

3. Nouwen EJ, Dauwe S, van der Biest I, De Broe ME. Stage- and segment-specific expression of cell-adhesion molecules N-CAM, A-CAM, and L-CAM in the kidney. *Kidney international* 1993, **44**(1)**:** 147-158.

4. Kroening S, Neubauer E, Wullich B, Aten J, Goppelt-Struebe M. Characterization of connective tissue growth factor expression in primary cultures of human tubular epithelial cells: modulation by hypoxia. *American journal of physiology Renal physiology* 2010, **298**(3)**:** F796-806.
