# Supplementary material for: Inhibition of MCL-1 to eliminate senescent cells and mitigate renal fibrosis in aristolochic acid nephropathy
Source: Cell Death Dis. 2025 Nov 26;17(1):56. doi: 10.1038/s41419-025-08268-7 (PMC12824375; doi:10.1038/s41419-025-08268-7)

Uncropped images for Western blot analysis

Figure 1

B

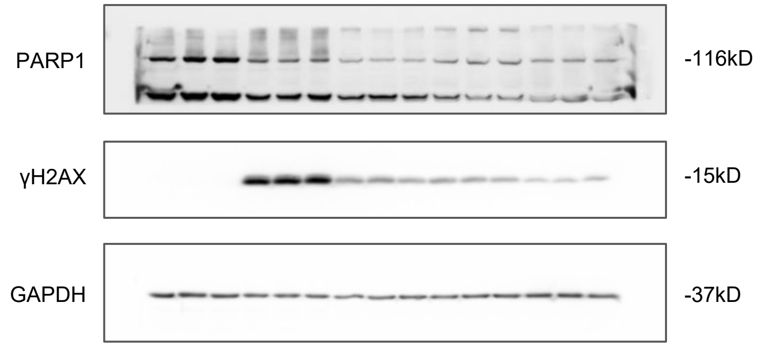

Figure 2

C

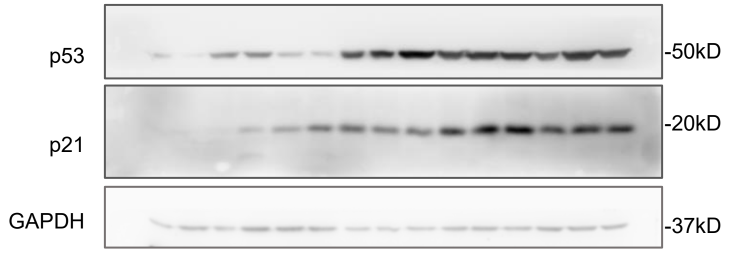

F

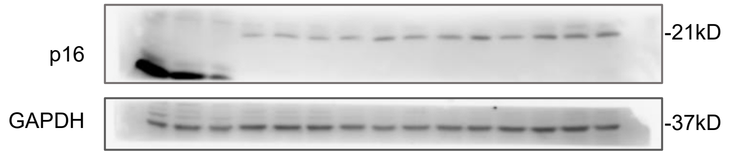

Figure 3

A

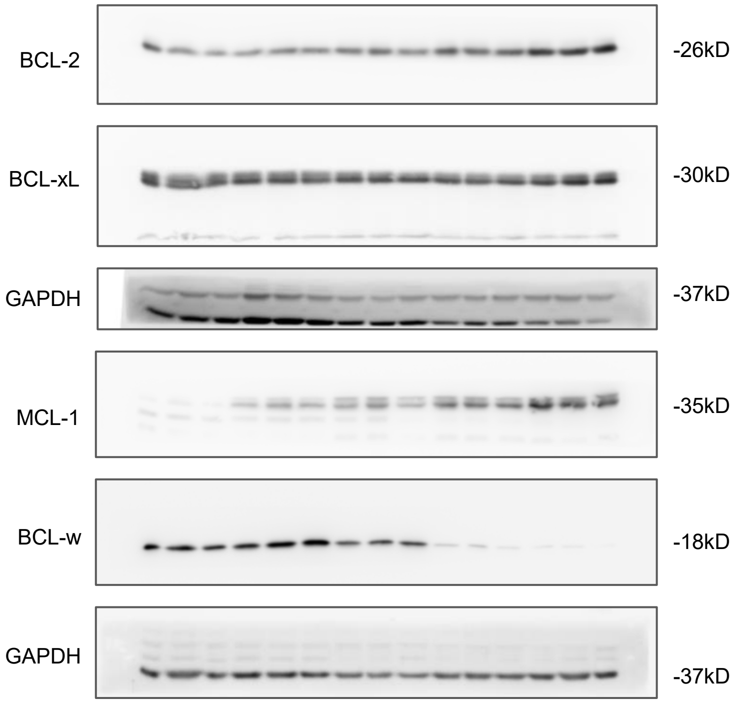

Figure 4

F

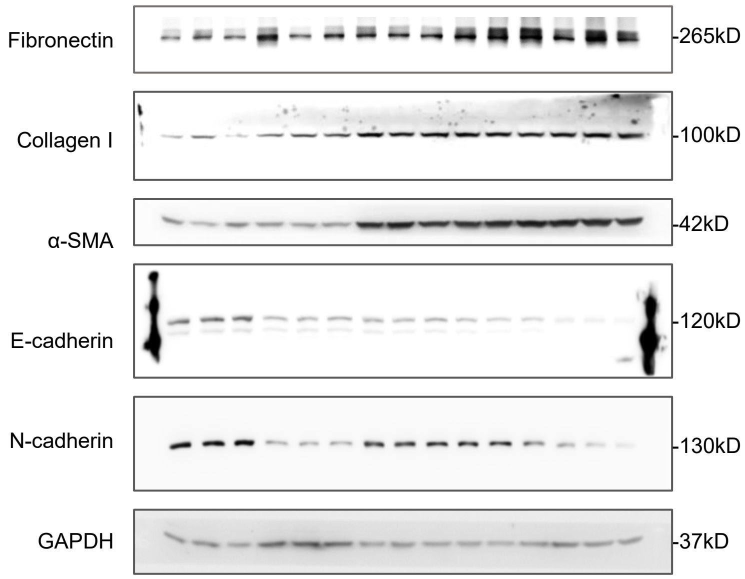

Figure 5

F

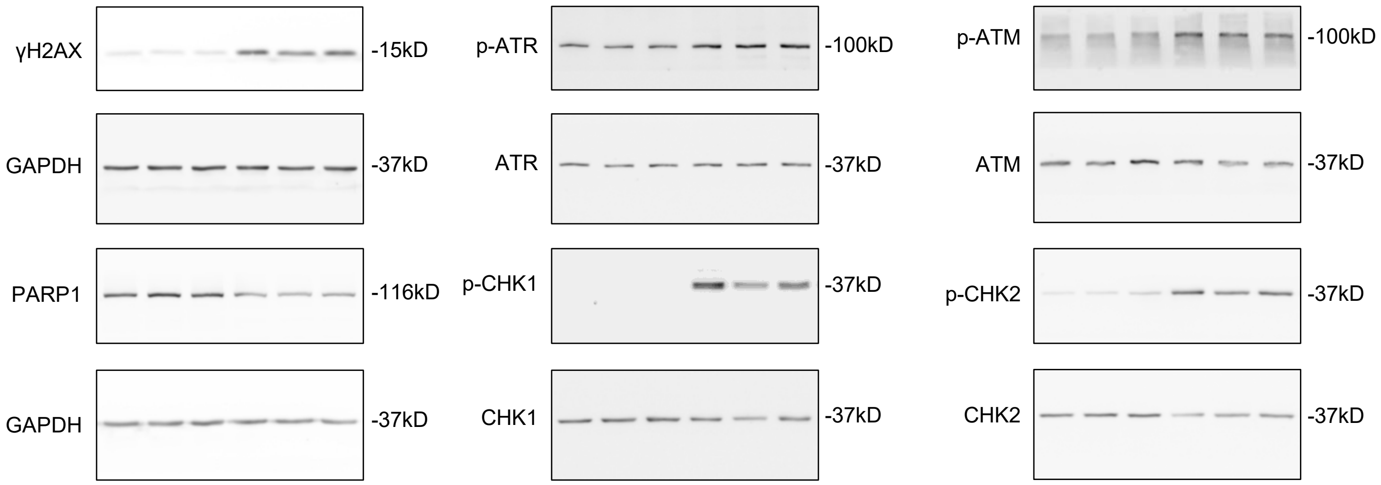

G

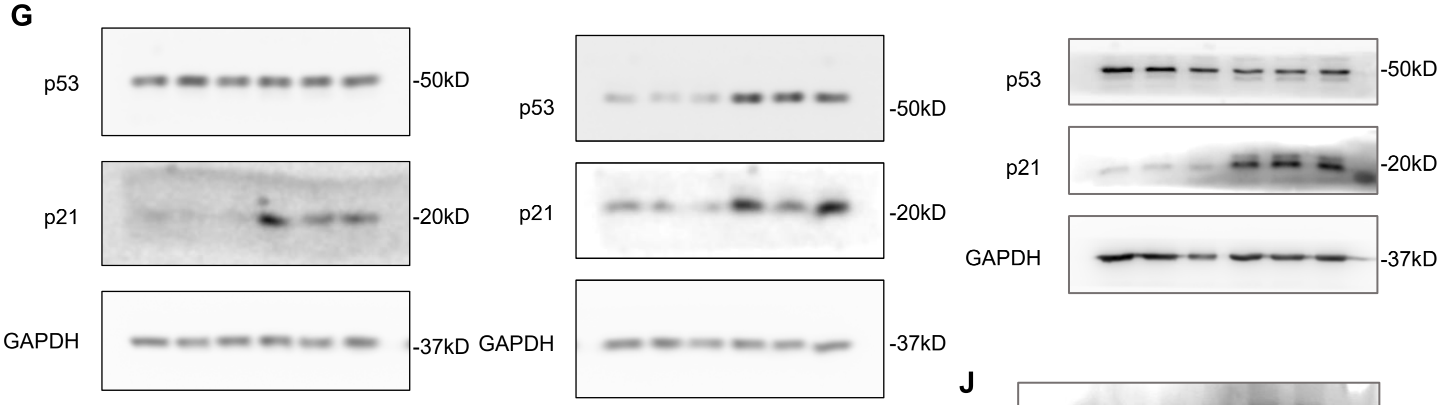

J

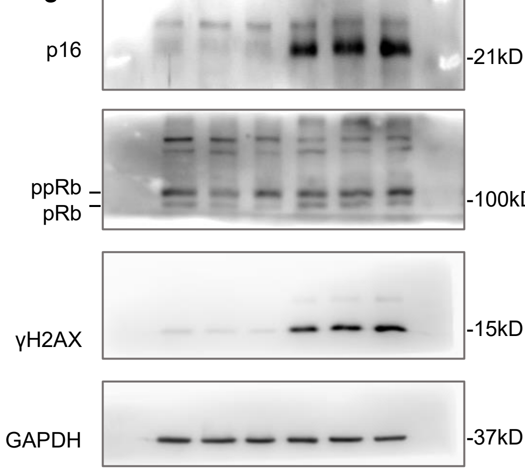

Figure 6

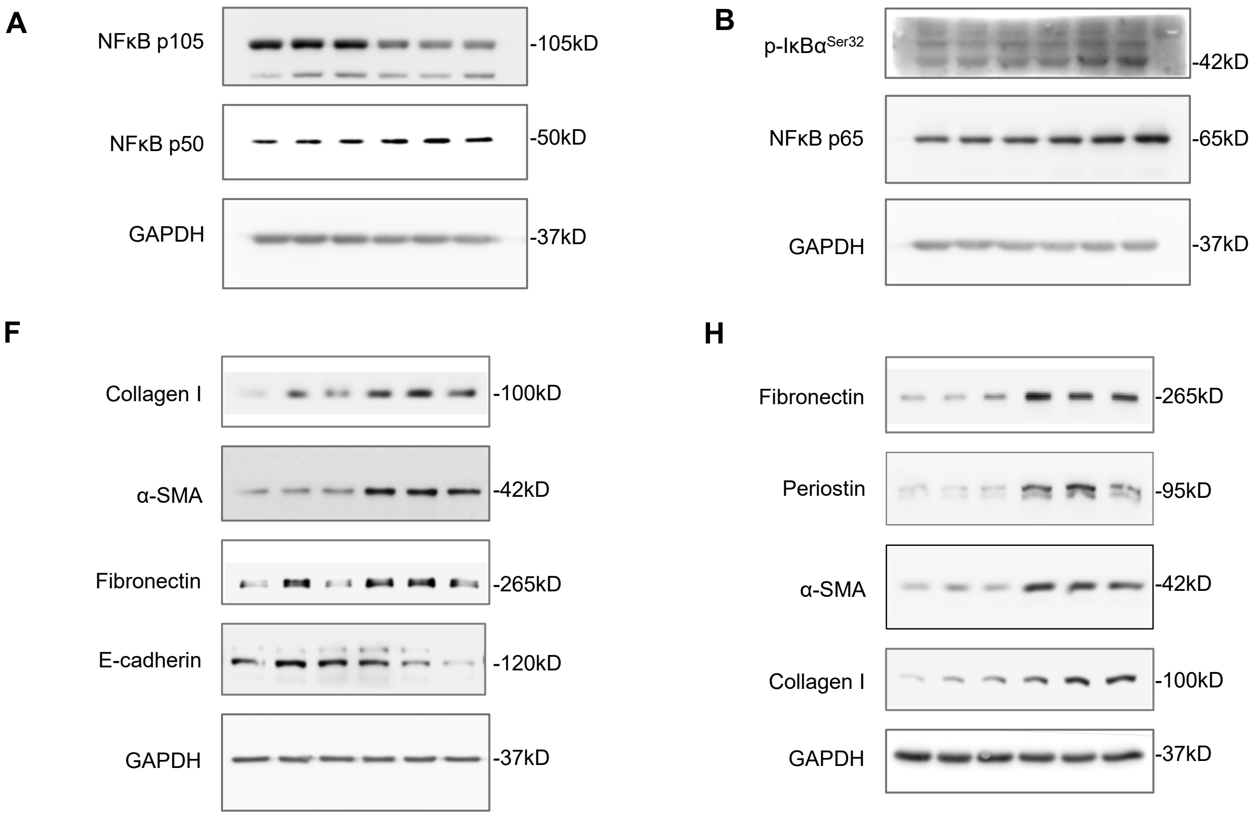

Figure 7

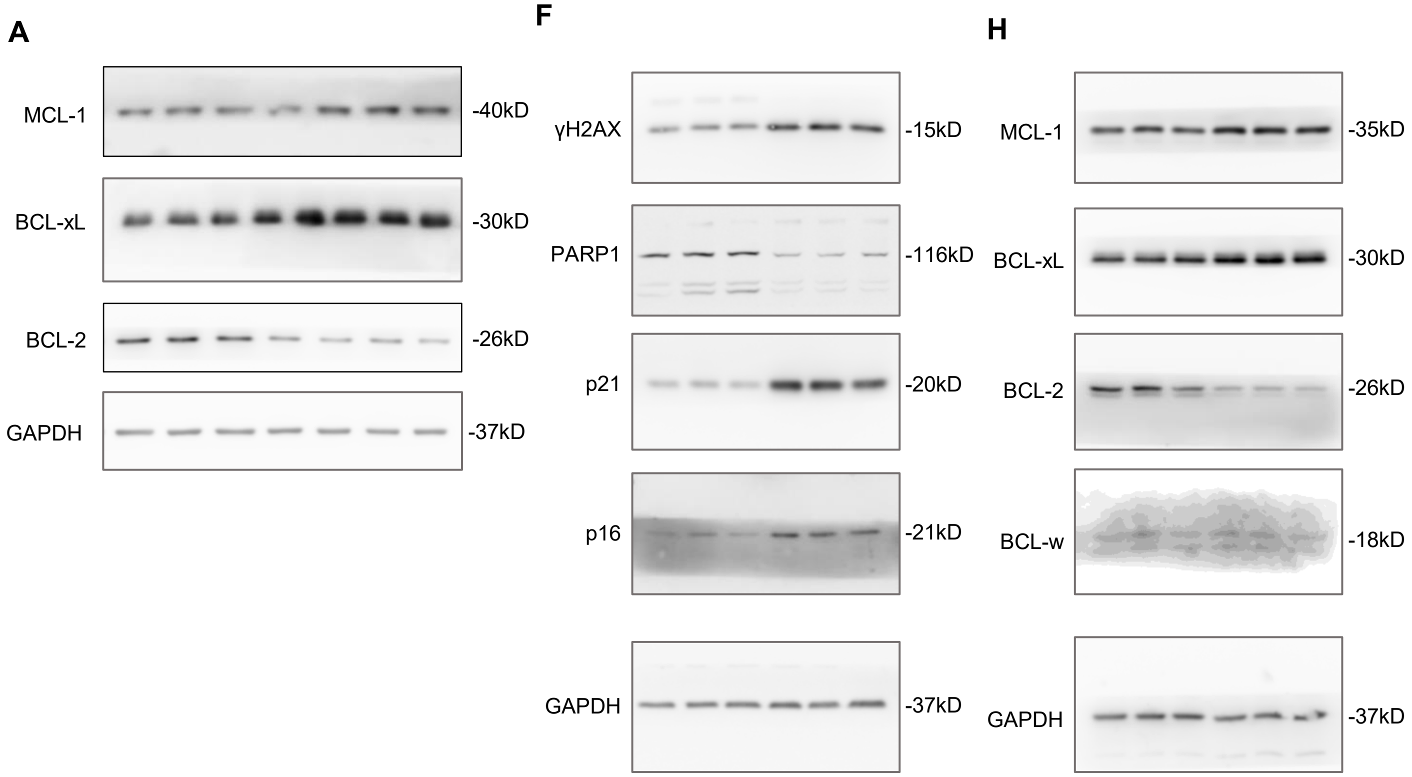

Figure 8

I

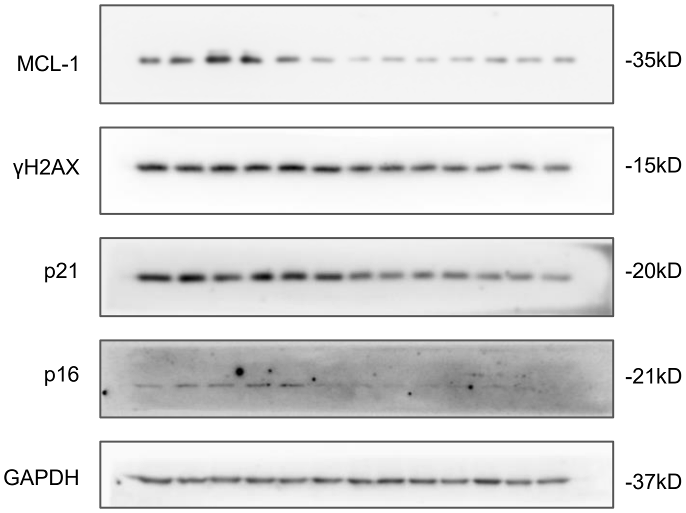

Figure 9

F

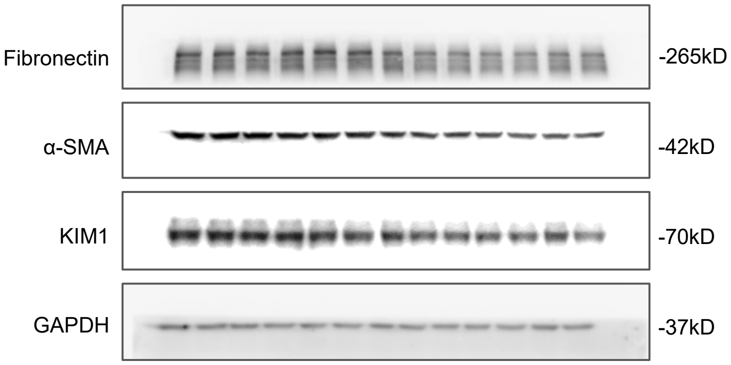

Supplementary Figure 2

A

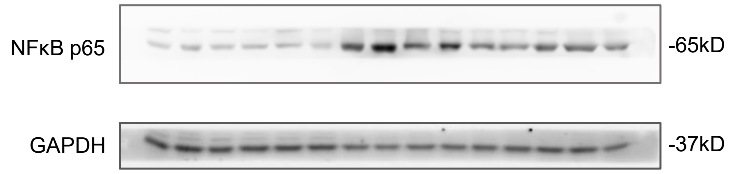

Supplementary Figure 3

C

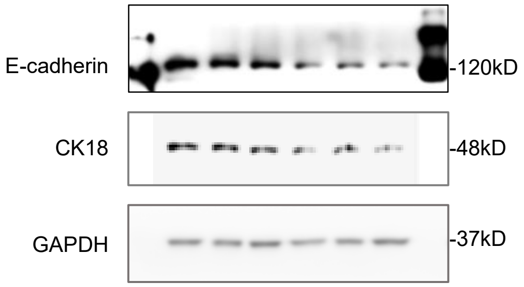

D

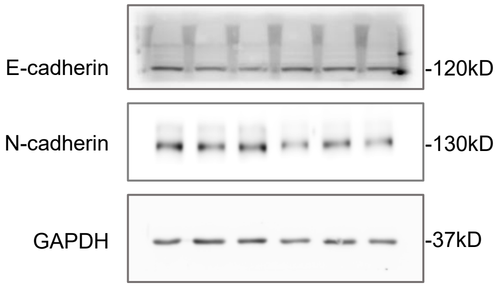

E

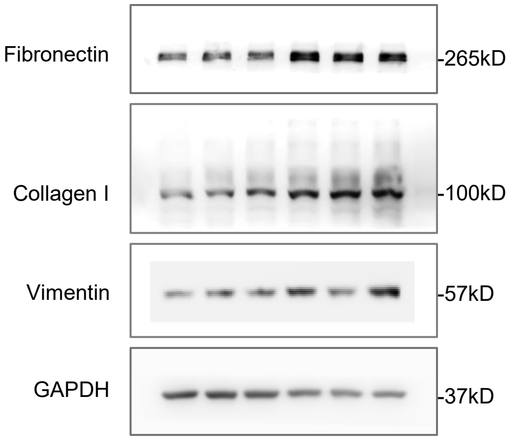

Supplementary Figure 9

C

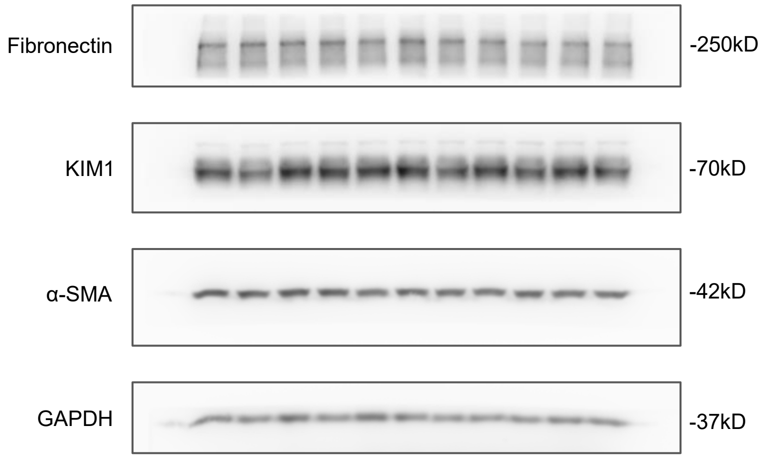

Supplementary Figure 10

D

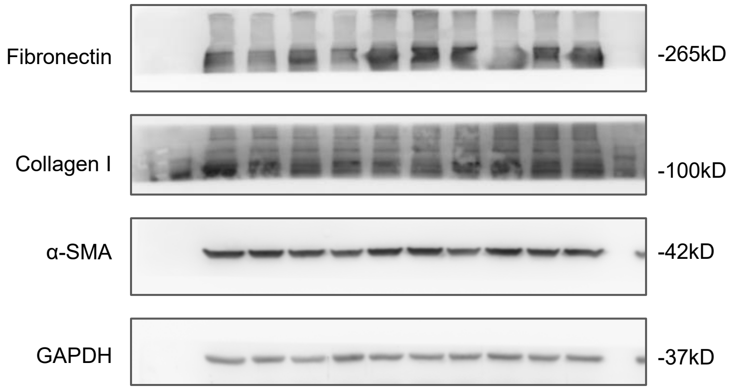

E

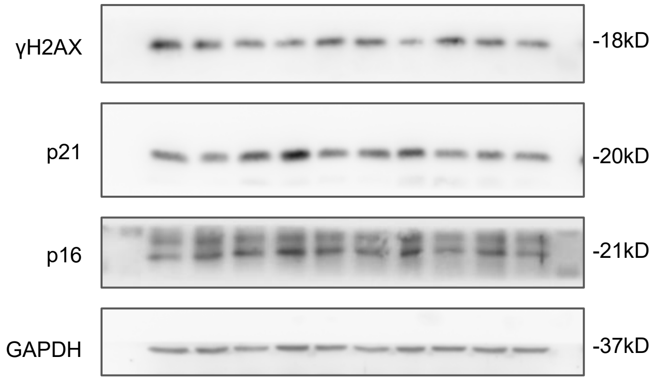

Supplementary Figure 11

C

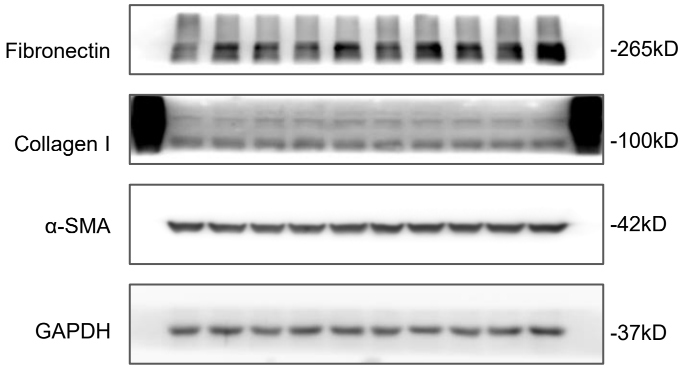

D

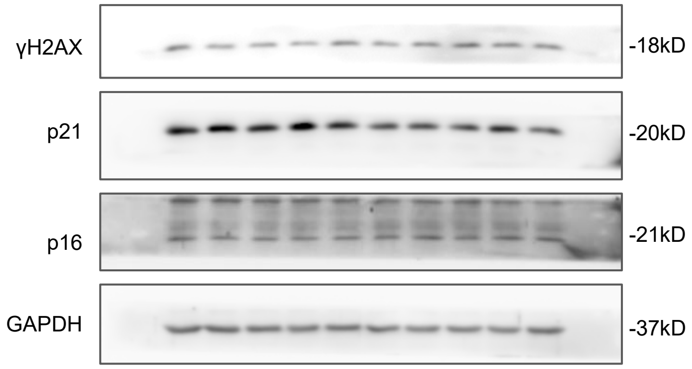

Supplementary Figure 12

C

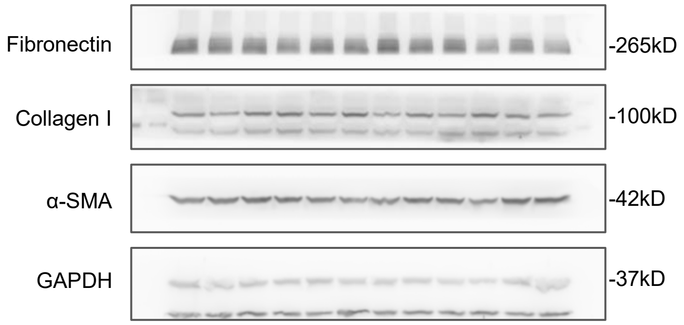

D

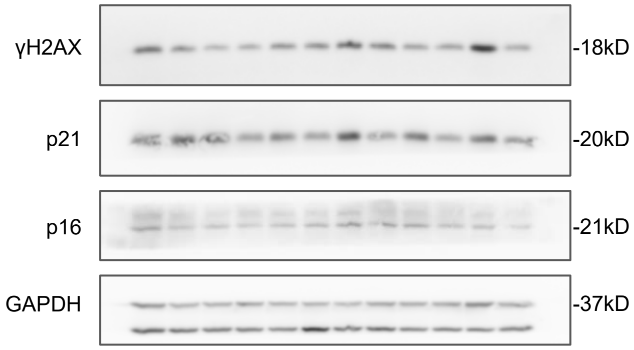

Supplementary Figure 14

I

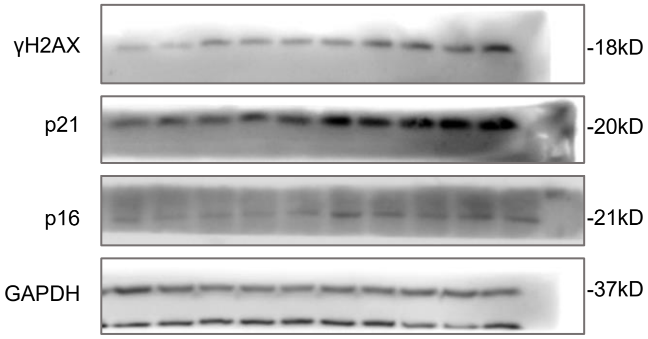

J

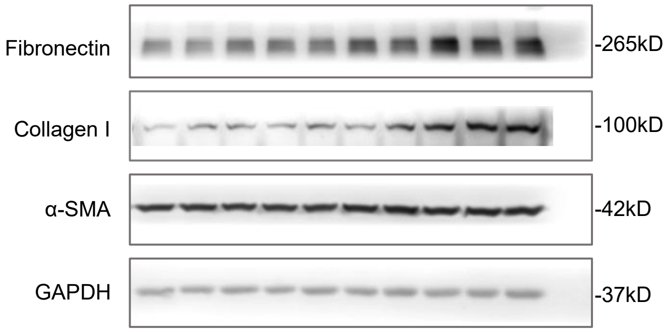

Supplement: Supplementary file 2 — Uncropped images for WB [file 41419_2025_8268_MOESM2_ESM.pdf]
